# IL-1 family signalling drives mucosal defence against systemic *Candida albicans* infection

**DOI:** 10.1038/s41564-026-02431-2

**Published:** 2026-08-05

**Authors:** James S. Griffiths, Alexander Kempf, Robert J. Pickering, Emily L. Priest, Olivia K. A. Paulin, Léa Lortal, Andrew Donkin, Olivia W. Hepworth, Don N. Wickramasinghe, Aize Pellon, Paul A. Stevens, Leanne Farnan, Adrian Lau, Harun Papini, Pawan Dhami, Sarah L. Gaffen, Jonathan P. Richardson, Julian R. Naglik

**Affiliations:** 1 Centre for Host–Microbiome Interactions, Faculty of Dentistry, Oral and Craniofacial Sciences, King’s College London, London, UK; 2 The Institute of Cancer Research, London, UK; 3 Department of Biochemistry and Biophysics, University of California San Francisco, San Francisco, CA USA; 4 Division of Infectious Diseases, Department of Medicine, Massachusetts General Hospital, Boston, MA USA; 5 Harvard Medical School, Boston, MA USA; 6 Inflammation and Macrophage Plasticity Laboratory, CIC bioGUNE-BRTA (Basque Research and Technology Alliance), Derio, Spain; 7 Genomics Research Platform R&D, Guy’s and St Thomas’ NHS Foundation Trust and King’s College London, London, UK; 8 Advanced Cytometry Platform (Flow Core), Research and Development Department at Guy’s and St Thomas’ NHS Foundation Trust, Guy’s Hospital, London, UK; 9 Division of Rheumatology and Clinical Immunology, University of Pittsburgh, Pittsburgh, PA USA

**Keywords:** Fungal host response, Fungal infection, Fungal infection, Risk factors

## Abstract

*Candida albicans* is a major opportunistic pathogen in humans that is capable of breaching mucosal barriers and causing severe systemic infections with high mortality. How the host controls mucosal infection and prevents dissemination remains unclear but is essential for improving disease outcomes. Here we demonstrate that *C. albicans* induces specific IL-1 family members, which are critical for initiating mucosal protection by controlling antimicrobial peptides, IL-17 and neutrophil responses. The loss of combined IL-1 family signalling led to severe oropharyngeal *C. albicans* infection, which was eventually resolved by a potent neutrophil response. However, in neutropenic conditions, a key patient risk factor, abolishing IL-1 family signalling resulted in *C. albicans* dissemination, predominantly to the liver, mirroring clinical disease and leading to mortality. This study highlights the IL-1 family as a key initiator of mucosal immunity, restricting mucosal invasion and cooperating with neutrophils to prevent life-threatening systemic infections.

## Abstract (web summary)

IL-1 family signalling drives host mucosal defence against *Candida albicans* and, together with neutrophils, prevents mucosal–systemic dissemination and life-threatening fungal disease.

## Main

Fungal pathogens are responsible for an increasingly severe global disease burden that kills ~2.5 million individuals every year. The fungus *Candida albicans* is a key contributor to this burden and is implicated in almost 1 million deaths annually^1^. *C. albicans* is typically a harmless constituent of a healthy microbiota but if left unchecked can overgrow its niche and drive superficial mucosal infection^2^. While mucosal infection is common and contributes to morbidity, it is invasive systemic disease that drives mortality. Systemic *C. albicans* infections arise from uncontrolled mucosal populations and from the colonization of medical plastics, coupled with ineffective host immunity^3,4^. Immunocompromised patients such as those with neutropenia (often a result of cancer treatment), graft versus host disease and HIV are at risk of invasive systemic disease^4^. With increasing resistance to antifungals, poor diagnostic tools and limited therapeutics, understanding how *C. albicans* mucosal infections develop and, critically, how they disseminate, is vital to managing *C. albicans* disease.

*C. albicans* is a polymorphic yeast that can produce invasive hyphal filaments that infect mucosal tissues. *C. albicans* hyphae secrete candidalysin, a toxin that damages mucosal barriers and activates innate host immune responses^5^. Mucosal tissues respond to the presence of candidalysin by inducing signal transduction pathways that promote the release of antimicrobial peptides (AMPs), proinflammatory cytokines, IL-17 (a key protective barrier cytokine) and recruit neutrophils^5–8^. In an immunocompetent host, this response usually resolves pathogenic fungal overgrowth, but immunocompromised hosts often struggle to contain the infection and develop superficial candidiasis. Uncontrolled mucosal candidiasis can penetrate through the mucosa resulting in invasive candidiasis, and ultimately disseminate to organs including the liver and spleen. However, while invasive disease is a severe clinical complication with high mortality (10–77%)^3,4,9^, not all susceptible patients develop invasive *C. albicans* infections, suggesting additional factors mediate invasion and dissemination.

The IL-1 family are a large cohort of cytokines, receptors and adaptor proteins that play a broad and essential role in host immunology. They are categorized into three subgroups comprising IL-1/IL-33, IL-18 and IL-36, with signalling typically occurring when cytokines bind their receptor, recruit a co-receptor and induce signalling through the Toll/interleukin-1 receptor domain, resulting in mitogen-activated protein kinase (MAPK) and nuclear factor kappa B (NF-κB) activation^10^. IL-1α and IL-1β are induced during mucosal *C. albicans* infection and mediate acute (day 1) neutrophil recruitment^11^ and drive the production of IL-17 (ref. ^12^). IL-36α/β/γ are also induced during mucosal *C. albicans* infection and mice lacking IL-36R exhibit enhanced susceptibility to mucosal disease^12^. While the protective effect of IL-36 is not fully understood, IL-36R deficiency reduced IL-23 expression, a cytokine important for the proliferation and survival of IL-17-producing cells^12,13^. IL-18 and IL-33 are induced by *C. albicans* infection^14,15^, though their roles in resolving mucosal disease remain unclear. In a mouse model of systemic candidiasis, IL-18 depletion increased disease susceptibility via an IFNγ–Th1 signalling axis^14^. Similarly, IL-33-deficient mice exhibited greater susceptibility to systemic infection, with IL-33 promoting IL-23 expression but downregulating IL-10 (ref. ^15^).

IL-1 family members are potent regulators of immunity, with insufficient activity frequently detrimental in a range of diseases but excessive activity being equally destructive^10,16^. Here, we investigated how the combinatorial IL-1 family shapes the host immune response to mucosal *C. albicans* infection and explored the role of the IL-1 family in mucosal–systemic dissemination. We found IL-1α/β, IL-33 and IL-36α/β/γ were induced at the mucosa following *C. albicans* infection. Using a murine model lacking the shared co-receptor for these agonists, the IL-1 receptor accessory protein (IL-1RAcP^‐/‐^), we demonstrate that early protective immunity during mucosal *C. albicans* infection is critically dependent on IL-1 family signalling. Remarkably, inducing neutropenia in IL-1RAcP^‐/‐^ mice, but not wild-type mice, resulted in dissemination of *C. albicans* to the liver and spleen (mimicking patient disease) and mortality. Our findings identify the IL-1 family as a key host risk factor for fungal dissemination and suggest that targeting IL-1 family signalling may prevent life-threatening invasive infections in immunocompromised patients.

## Results

### The IL-1 family are rapidly induced at the mucosa and control fungal infection

**Fig. 1:**
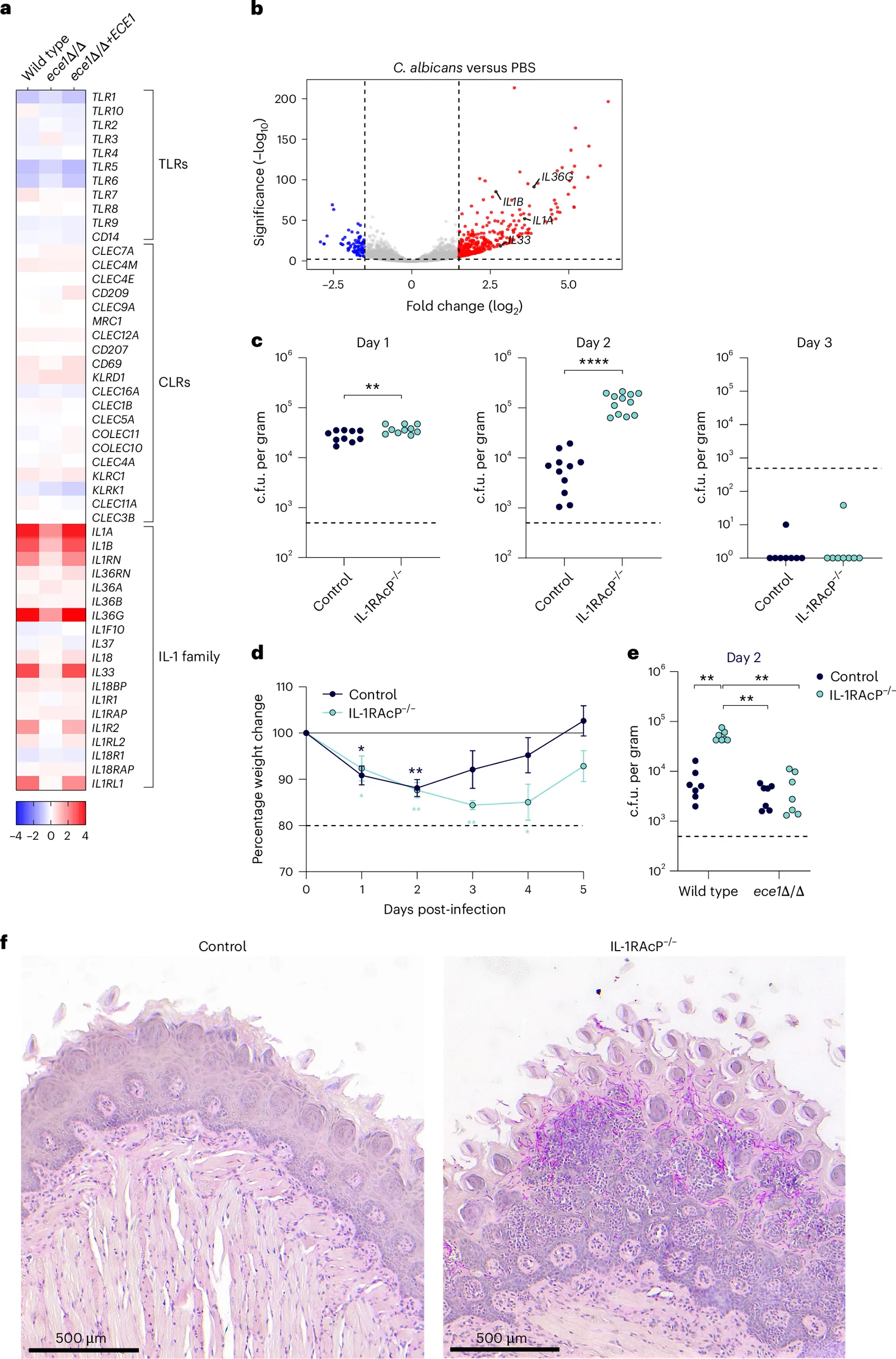
The IL-1 family are rapidly induced by candidalysin and restrict *C. albicans* infection and invasion. **a**,**b**, TR146 cells were challenged with wild-type *C. albicans* (BWP17 + CIp30), *ece1*Δ/Δ or *ece1*Δ/Δ+*ECE1* before bulk RNA sequencing was performed. Selected constituents of TLR, CLR and IL-1 family signalling pathways were identified^10,77^ (and KEGG pathway reference hsa04620) and relative gene expression data displayed as a heat map (**a**). Total bulk RNA gene expression data are displayed as a volcano plot according to their differential expression when compared with PBS (**b**). **c**,**d**, Control (C57BL/6J) and IL-1RAcP^‐/‐^ mice were sublingually infected with wild-type *C. albicans* (AHY940). The c.f.u. was enumerated from tongue homogenate and analysed by a Kruskal–Wallis test with Dunn’s post-test (**c**). Each symbol represents one mouse (day 1, *n* = 10 for each group; day 2, *n* = 11 control and *n* = 12 IL-1RAcP^‐/‐^; day 3, *n* = 8 for each group); **P = 0.0085, ****P < 0.0001. The dotted line represents the limit of detection. Weight change of grouped control and IL-1RAcP^‐/‐^ mice data from three independent experiments (*n* = 10 for each group) (**d**). Each timepoint was compared with the day 0 value by two-way ANOVA with Bonferroni’s post-test. Control: day 1, *adj. *P* = 0.0429; day 2, **adj. *P* = 0.0031. IL-1RAcP^‐/‐^: day 1 *adj. *P* = 0.0433, day 2 **adj. *P* = 0.0072, day 3 **adj. *P* = 0.0055, day 4 *adj. *P* = 0.0163. Data presented as mean ± s.e.m. The dotted line represents the experimental limit. **e**, Control and IL-1RAcP^‐/‐^ mice were sublingually infected with *C. albicans* (AHY940) or *ece1*Δ/Δ. The c.f.u. was enumerated from tongue homogenate and analysed by two-way ANOVA with Bonferroni’s post-test. Each symbol represents one mouse (*n* = 8 for each group). Wild-type control versus IL-1RAcP^‐/‐^ **adj. *P* = 0.0026; wild-type IL-1RAcP^‐/‐^ versus *ece1*Δ/Δ control **adj. *P* = 0.0042, wild-type IL-1RAcP^‐/‐^ versus *ece1*Δ/Δ IL-1RAcP^‐/‐^ **adj. *P* = 0.0033. The dotted line represents the limit of detection. **f**, Control and IL-1RAcP^‐/‐^ mice were sublingually infected with *C. albicans* (AHY940) before tongue tissue was collected on day 2 and periodic acid–Schiff stained. Representative images are shown, similar results were obtained in three independent experiments (*n* = 3). Statistical significance: adjusted (adj.) *P < 0.05, **P < 0.01, ***P < 0.001, ****P < 0.0001. Source data

**Extended Data Fig. 1:**
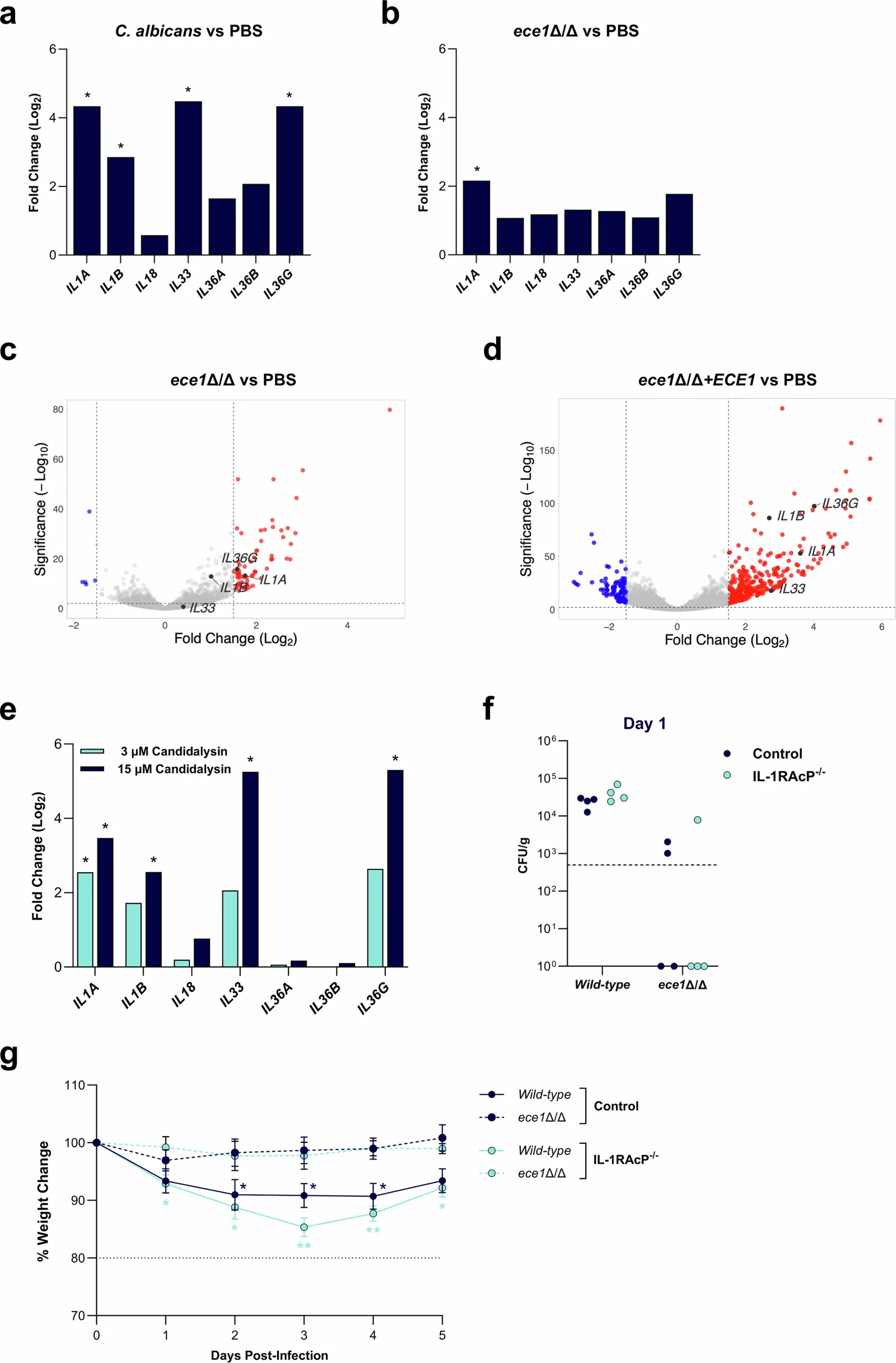
C. albicans and candidalysin drive mucosal infection and IL-1 family responses. (**a-d**) TR146 cells were challenged with wild-type *C. albicans* (BWP17 + CIp30) or *ece1*Δ/Δ before bulk RNA sequencing was performed. (**a-b**) Log_2_FC values for IL-1 family members were identified after challenge with either *C. albicans* (BWP17 + CIp30) or *ece1*Δ/Δ and their significance analysed using R package ‘DESeq2’ and Wald test p value (FDR < 0.01).(**a**) *IL1A* *adj.*P* = 3.5 ×10^‒53^, *IL1B* *adj.*P* = 3.24 ×10^‒86^, *IL33* *adj.*P* = 2.1 ×10^‒19^, *IL36G* *adj.*P* = 4 ×10^‒92^. (**b**) *IL1A* *adj.*P* = 7.13 ×10^‒13^. (**c-d**) Total bulk RNA gene expression results for *ece1*Δ/Δ or *ece1*Δ/Δ+*ECE1* displayed as a volcano plot according to their differential expression when compared to PBS. (**e**) TR146 cells were challenged with 3 µM or 15 µM candidalysin before bulk RNA sequencing was performed. Log_2_FC values for IL-1 family members were calculated and their significance analysed using R package ‘DESeq2’ and Wald test p value (FDR < 0.01). 3 µM: *IL1A* *adj.*P* = 8.5 ×10^‒53^. 15 µM: *IL1A* *adj.*P* = 9.81 ×10^‒94^, *IL1B* *adj.*P* = 4.53 ×10^‒22^, *IL33* *adj.*P* = 4.01 ×10^‒75^, *IL36G* *adj.*P* = 4.66 ×10^‒133^. (**f-g**) Control and IL-1RAcP^‐/‐^ mice were sublingually infected with *C. albicans* (AHY940) or *ece1*Δ/Δ. (**f**) CFU was enumerated from tongue homogenate and analysed by 2-way ANOVA with Bonferroni’s post-test. Each symbol represents one mouse (n = 4 for each group). (**g**) Weight change of grouped control and IL-1RAcP^‐/‐^ mice data from 2 independent experiments (n = 7 for each group). Each timepoint was compared to the day 0 value by 2-way ANOVA with Bonferroni’s post-test. Wild-type Control: day 2 *adj.*P* = 0.0102, day 3 *adj.*P* = 0.0119, day 4 *adj.*P* = 0.0279. Wild-type IL-1RAcP^‐/‐^: day 1 *adj.*P* = 0.0196, day 2 *adj.*P* = 0.0214, day 3 **adj.*P* = 0.0016, day 4 **adj.*P* = 0.0026, day 5 *adj.*P* = 0.0193. Data presented as mean ± s.e.m. Black dotted line represents experimental limit. Statistical significance: adjusted (adj.), *P < 0.05, **P < 0.01. Source data

To identify which IL-1 family members and immune recognition pathways were induced during *C. albicans* mucosal infection, we challenged TR146 human oral epithelial cells with *C. albicans* BWP17 + CIp30 (wild-type; derivative of SC5314) and performed RNA sequencing. At 4 h post-infection, we observed the induction of *IL1A*, *IL1B*, *IL33* and *IL36G* but not toll-like receptor (TLR) and C-type lectin (CLR) receptor pathways (Fig. 1a and Extended Data Fig. 1a). Notably, IL-1 family members were some of the most significantly upregulated genes (Fig. 1b). Given the rapid induction of IL-1 family signalling, and with IL-1α being a damage-associated cytokine, we hypothesized that IL-1 family induction was dependent on the secretion of the damage-inducing peptide toxin candidalysin from *C. albicans*^5^. To test this, RNA sequencing was performed on TR146 cells infected with a *C. albicans* strain deficient in candidalysin (*ece1*Δ/Δ) and a re-integrant control (*ece1*Δ/Δ*+ECE1*), as well as synthetic candidalysin at weakly and strongly stimulating concentrations (3 µM and 15 µM, determined previously^5^). The induction of IL-1 family signalling was critically dependent on candidalysin (Fig. 1a and Extended Data Fig. 1b–d) and the expression of IL-1 family members increased in a candidalysin concentration-dependent manner (Extended Data Fig. 1e).

IL-1α, IL-1β, IL-33 and IL-36γ, the *C. albicans*-induced members of the IL-1 family, signal through a complex of their primary receptor (IL-1R, ST2 and IL-36R) and their common co-receptor, the IL-1 receptor accessory protein (IL-1RAcP). Therefore, we used an IL-1RAcP^‐/‐^ murine model to investigate the impact of abolishing combinatorial IL-1 family signalling during *C. albicans* oral infection. We sublingually challenged IL-1RAcP^‐/‐^ and control (C57BL/6J) mice with *C. albicans* AHY940 (wild type, derivative of SC5314) to induce oropharyngeal candidiasis (OPC), collected tongue tissue and quantified fungal burden (colony forming units (c.f.u.)). IL-1RAcP^‐/‐^ mice failed to initially control the infection and displayed a remarkably acute susceptibility to a typically mild OPC challenge, as demonstrated by significantly higher fungal burdens at day 1 and most notably at day 2 (Fig. 1c). Despite this, both control and IL-1RAcP^‐/‐^ mice cleared the infection by day 3. Weight change data corroborated the severity of the infection with IL-1RAcP^‐/‐^ mice experiencing significant weight loss for 4 days post infection (nearing the 80% experimental limit) before recovering (Fig. 1d).

Given that the severity of OPC was dependent on IL-1 family signalling and IL-1 family induction was dependent on candidalysin, we next investigated whether candidalysin was driving virulence in the IL-1RAcP^‐/‐^ model. We challenged IL-1RAcP^‐/‐^ and control mice with *C. albicans* (AHY940) and *ece1*Δ/Δ to induce OPC and quantified c.f.u. in tongue tissue. Notably, *ece1*Δ/Δ showed significantly reduced burdens in both control and IL-1RAcP^‐/‐^ mice (Fig. 1e and Extended Data Fig. 1f), and no weight loss in either model (Extended Data Fig. 1g). This confirmed that candidalysin was driving *C. albicans* virulence in the IL-1RAcP^‐/‐^ model.

We next examined mucosal tissue to investigate the extent of *C. albicans* invasion. Histological sections of tongue tissue were taken from IL-1RAcP^‐/‐^ and control mice 2 days post-OPC and analysed with periodic acid–Schiff staining. While control mice had little detectable *C. albicans* and an intact mucosal barrier, IL-1RAcP^‐/‐^ mice exhibited a heavily disrupted mucosal barrier with large quantities of *C. albicans* hyphae invading deep into the mucosa (Fig. 1f). Our data demonstrate that combined IL-1 family signalling is critically important during early OPC and deficiencies in IL-1 signalling radically enhance susceptibility to *C. albicans* infection.

### Mucosal AMPs and IL-17 expression require IL-1 family signalling

**Fig. 2:**
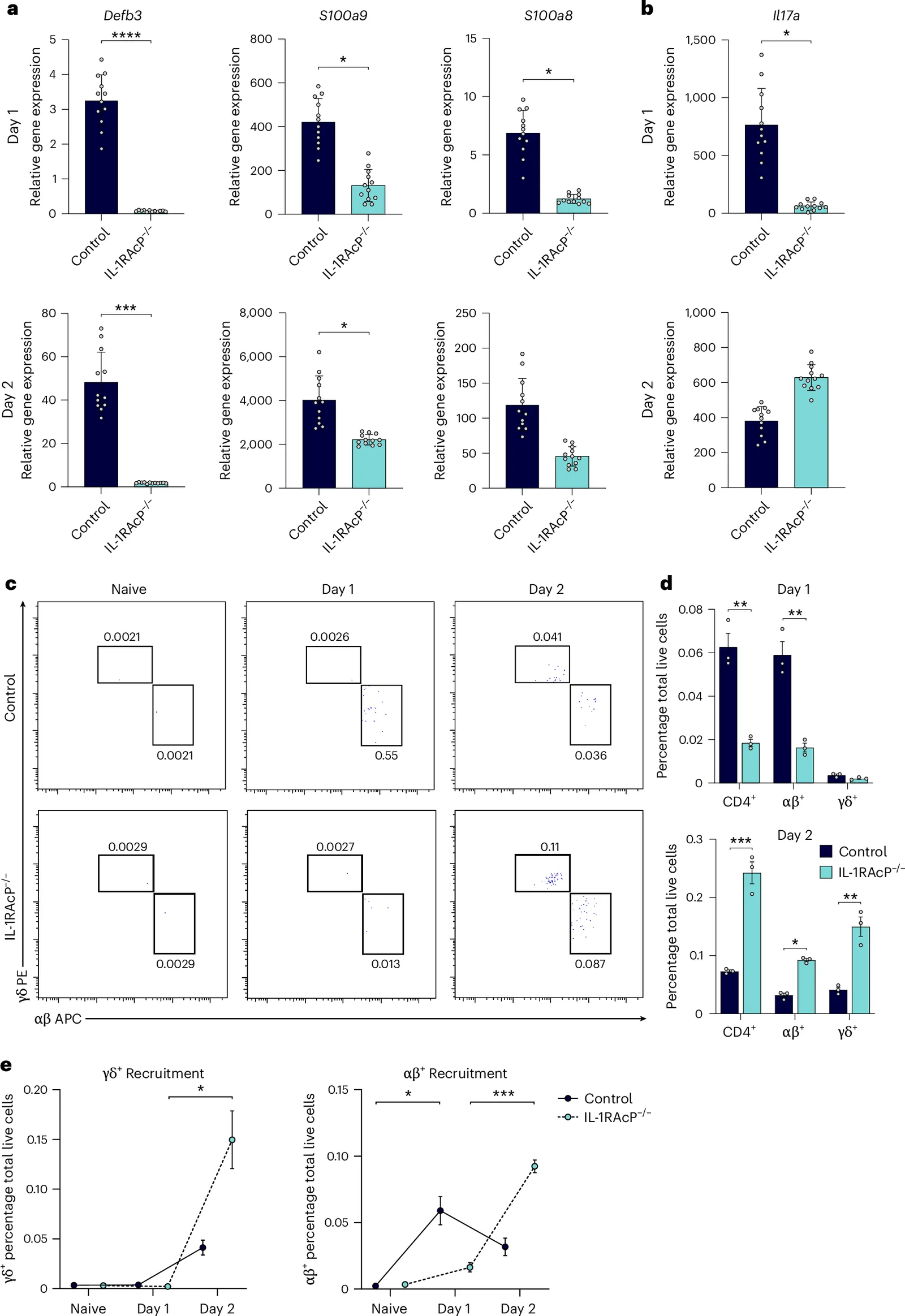
The IL-1 family initiate protective mucosal barrier immunity. **a**−**e**, Control and IL-1RAcP^‐/‐^ mice were sublingually infected with *C. albicans* (AHY940). **a**,**b**, RNA was extracted from tongue and gene expression determined by RT–qPCR (**a** and **b**). The results were quantified by ΔΔ*Ct* analysis compared against a control naive sample. Differences between control and IL-1RAcP^‐/‐^ mice were analysed by a Kruskal–Wallis test with Dunn’s post-test. The graph displays cumulative data from at least three independent experiments (*n* = 12 for each group). *Defb3* day 1 ****P < 0.0001, day 2 ***P = 0.0006; *S100a9* day 1 *P = 0.0108, day 2 *P = 0.0286; *S100a8* day 1 *P = 0.0286; *Il17a* day 1 *P = 0.0286. Data are presented as median with interquartile range. In **c**–**e**, tongue tissue was homogenized to single cells and stained with a live/dead dye, anti-CD45, anti-CD4, anti-TCRαβ and anti-TCRγδ antibodies and/or their appropriate isotypes before being analysed by flow cytometry. Flow plots representative of data from at least three independent experiments, gating shown as live (dead^+^ exclusion), single, CD45^+^, CD4^+^ and as percentage of total live cells (**c**). CD4^+^, αβ^+^ and γδ^+^ cells were quantified from at least three independent experiments (*n* = 3 for each group) and differences between control and IL-1RAcP^‐/‐^ analysed by two-way ANOVA with Bonferroni’s post-test (**d**). Day 1: CD4^+^ **adj. *P* = 0.0028, αβ^+^ **adj. *P* = 0.0045; day 2 CD4^+^ ***adj. *P* = 0.0002, αβ^+^ *adj. *P* = 0.0102, γδ^+^ **adj. *P* = 0.0018. Data presented as mean ± s.e.m. αβ^+^ and γδ^+^ cells (as % total live cells) from at least three independent experiments (*n* = 3 for each group) were tracked from naive to day 2 of infection (**e**). Differences within each group between each timepoint were analysed by two-way ANOVA with Bonferroni’s post-test. γδ^+^ *P = 0.0236, αβ^+^ *P = 0.0130, ***P = 0.0004. Data presented as mean ± s.e.m. Statistical significance: adj. *P < 0.05, **P < 0.01, ***P < 0.001, ****P < 0.0001. Source data

**Extended Data Fig. 2:**
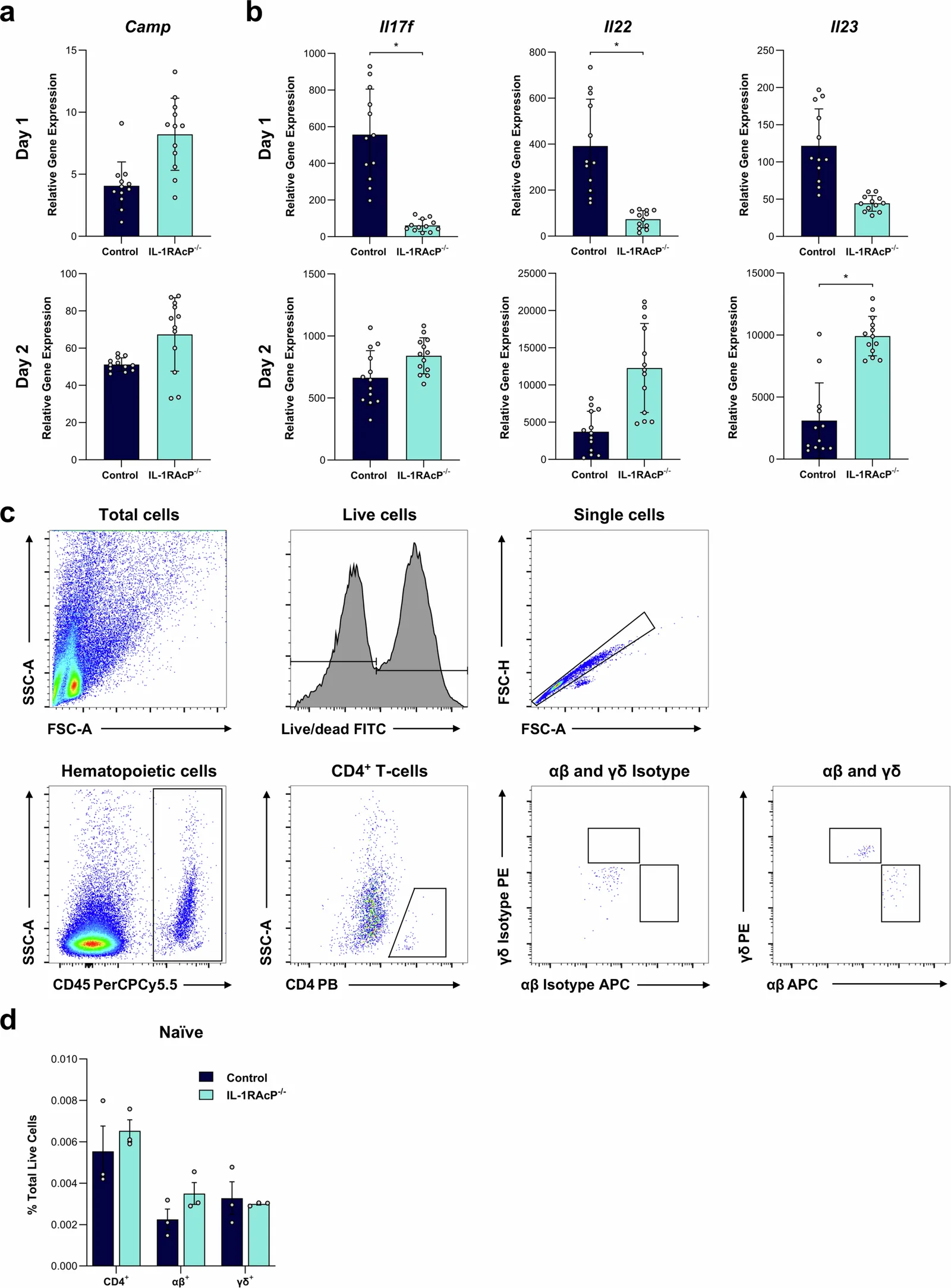
IL-1 family signalling regulates mucosal cytokine responses and T-cell populations. (**a-c**) Control and IL-1RAcP^‐/‐^ mice were sublingually infected with *C. albicans* (AHY940). (**a-b**) RNA was extracted from tongue and gene expression determined by RT-qPCR. Results were quantified by *ΔΔCt* analysis compared against a naïve sample. Differences between control and IL-1RAcP^‐/‐^ were analysed by Kruskal-Wallis test with Dunn’s post-test. *Il17f* *P = 0.0298, *Il22* *P = 0.0417, *Il23* *P = 0.0107. Graph displays cumulative data from at least 3 independent experiments (n = 12 for each group). Data presented as median with interquartile range (**c-d**) Tongue tissue was homogenised to single cells and stained with a live/dead dye, anti-CD45, anti-CD4, anti-TCRαβ and anti-TCRγδ antibodies and/or their appropriate isotypes before being analysed by flow cytometry. (**c**) Gating strategy for isolating CD4^+^, αβ^+^ and γδ^+^ cells completed on day 2 post-OPC IL-1RAcP^‐/‐^ sample. (**d**) CD4^+^, αβ^+^ and γδ^+^ cells were quantified from at least 3 independent experiments (n = 3 for each group) and differences between control and IL-1RAcP^‐/‐^ analysed by 2-way ANOVA with Bonferroni’s post-test. Data presented as mean ± s.e.m. Statistical significance: *P < 0.05. Source data

The production of AMPs and induction of innate type 17 immunity is critical for barrier immunity in acute OPC infections^6,13,17^. Thus, we investigated whether the high susceptibility of IL-1RAcP^‐/‐^ mice to OPC was associated with impairments in the T_H_17 axis. We induced OPC in IL-1RAcP^‐/‐^ and control mice, collected mucosal tissue and quantified β-defensin 3 (*Defb3*), calprotectin (a heterodimer of *S100a8* and *S100a9*), *Il17a/f*, *Il22* and *Il23a* gene expression. We found abolishment of β-defensin 3 expression and significantly reduced calprotectin expression at both day 1 and day 2 post infection in IL-1RAcP^‐/‐^ mice (Fig. 2a). Given that β-defensin 3 is predominantly epithelial derived^18^, this suggests that β-defensin 3 expression is dependent on the IL-1 family–IL-17 axis during OPC. We also examined cathelicidin (*Camp*) but found no IL-1 family dependency (Extended Data Fig. 2a). *Il17a/f*, *Il22* and *Il23a* expression was largely dependent on IL-1 family signalling at day 1, but importantly recovered by day 2 (Fig. 2b and Extended Data Fig. 2b), even with a fungal burden ten-times greater in IL-1RAcP^‐/‐^ mice (Fig. 1c).

The production of IL-17 during acute OPC is predominantly mediated by innate-acting TCRαβ^+^ and TCRγδ^+^ cells^13,19^. Therefore, we induced OPC in IL-1RAcP^‐/‐^ and control mice and assessed the infiltration and expansion of TCRαβ^+^ and TCRγδ^+^ cells. While naive (uninfected) mice exhibited no difference in cellular infiltration (Extended Data Fig. 2c,d), the acute infiltration (day 1) of TCRαβ^+^ cells was IL-1 family dependent (Fig. 2c,d). However, by day 2, the infiltration of both TCRαβ^+^ and TCRγδ^+^ cells had fully recovered to levels far greater than control mice (Fig. 2d,e), indicating that cellular recruitment once infection was established was IL-1 family independent. These data suggest that early (day 1) protective barrier immune responses in OPC require combined IL-1 family signalling, and deficiency permits *C. albicans* overgrowth and increased virulence.

### Mucosal neutrophil infiltration is delayed without IL-1 family signals

**Fig. 3:**
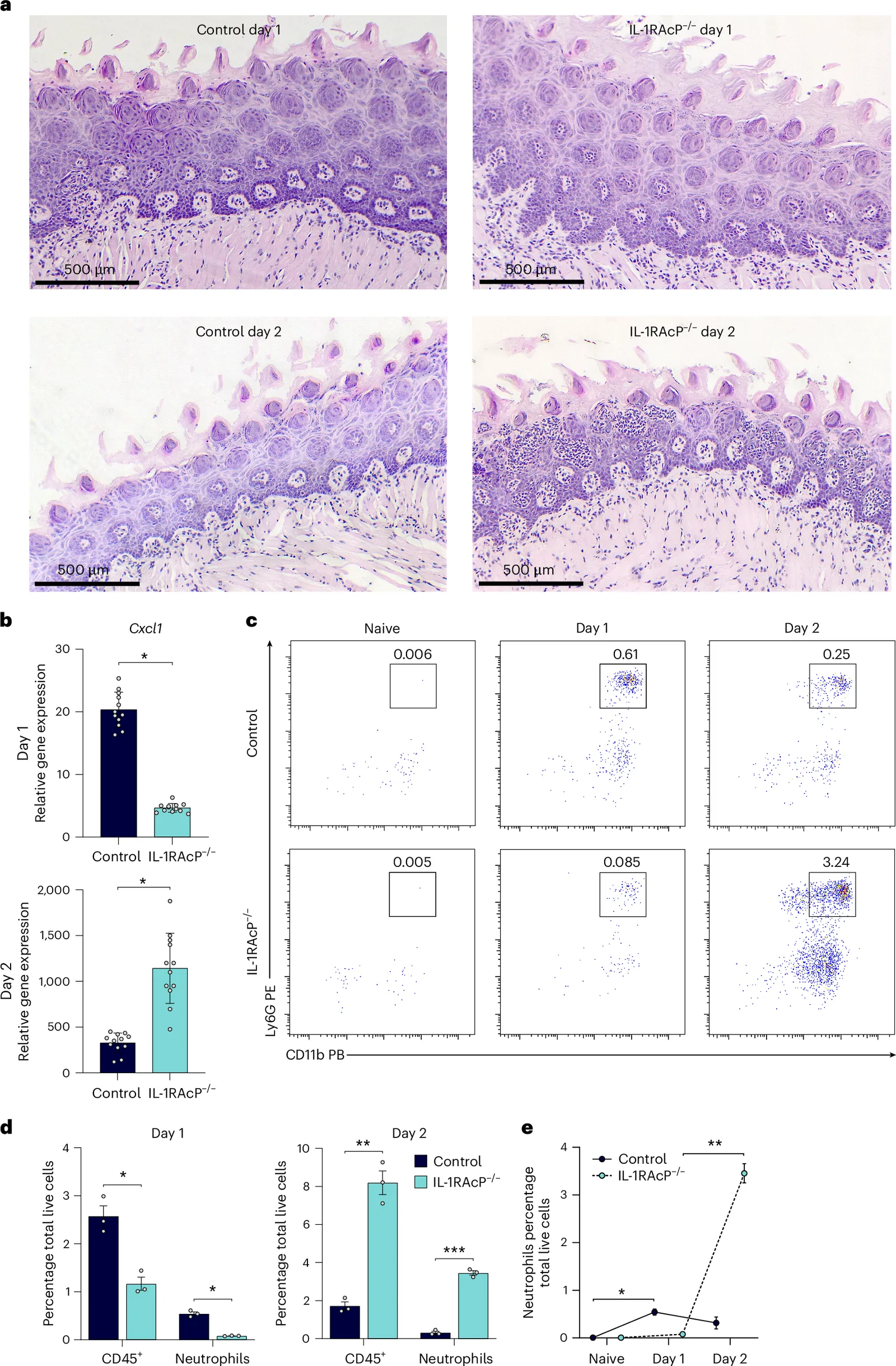
Rapid mucosal neutrophil recruitment is dependent on IL-1 family signalling. Control and IL-1RAcP^‐/‐^ mice were sublingually infected with *C. albicans* (AHY940). **a**, Tongue tissue was collected and stained with H&E. Representative images are shown, similar results were obtained in three independent experiments (*n* = 3). **b**, RNA was extracted from tongue and gene expression determined by RT–qPCR. Results were quantified by ΔΔ*Ct* analysis compared against a control naive sample. Graph displays cumulative data from at least three independent experiments (*n* = 12 for each group). Differences between control and IL-1RAcP^‐/‐^ were analysed by a Kruskal–Wallis test with Dunn’s post-test. *Cxcl1* day 1 *P = 0.0286, day 2 *P = 0.0289. Data are presented as median with interquartile range. **c**, Tongue tissue was homogenized to single cells and stained with a live/dead dye, anti-CD45, anti-Ly6G and anti-CD11b antibodies and/or their appropriate isotypes before being analysed by flow cytometry. Flow plots representative of data from at least three independent experiments, gating shown as live (dead^+^ exclusion), single, CD45^+^ and as percentage of total live cells. **d**, CD45^+^ and CD45^+^Ly6G^+^CD11b^+^ cells were quantified from at least three independent experiments (*n* = 3 for each group). Differences between control and IL-1RAcP^‐/‐^ were analysed by two-way ANOVA with Bonferroni’s post-test. Day 1: CD45^+^ *adj. *P* = 0.022, neutrophils *adj. *P* = 0.0102; day 2 CD45^+^ **adj. *P* = 0.0045, neutrophils ***adj. *P* = 0.0004. Data presented as mean ± s.e.m. **e**, CD45^+^ and CD45^+^Ly6G^+^CD11b^+^ cells (as percentage total live cells) from at least three independent experiments (*n* = 3 for each group) were tracked from naive to day 2 of infection. Differences within each group at each timepoint were analysed by two-way ANOVA with Bonferroni’s post-test. *adj. *P* = 0.0482, **adj. *P* = 0.0083. Data presented as mean ± s.e.m. Statistical significance: adj. *P < 0.05, **P < 0.01, ***P < 0.001, ****P < 0.0001. Source data

**Extended Data Fig. 3:**
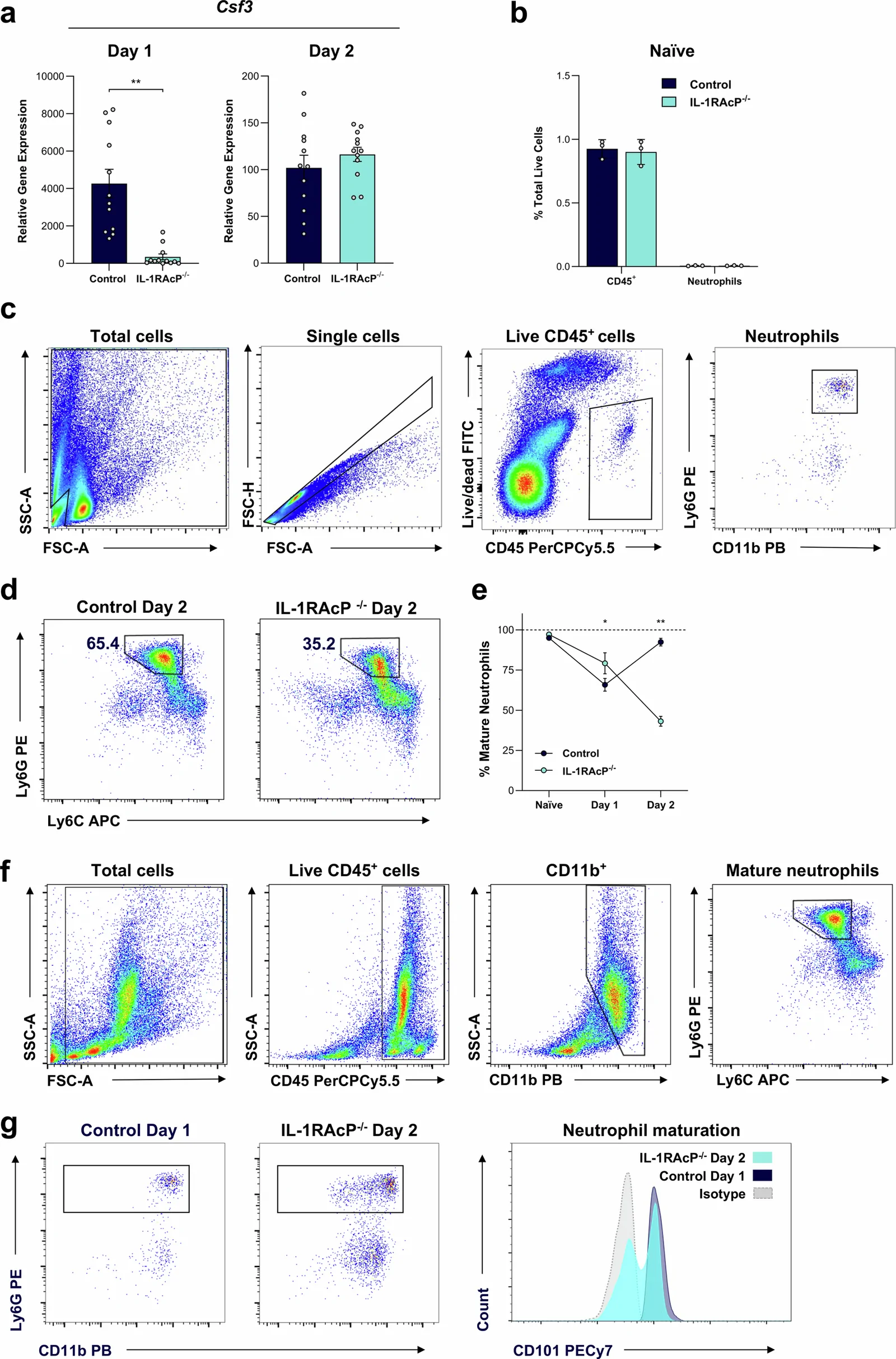
Neutrophil infiltration is partially controlled by IL-1 family signalling. (**a**) Control and IL-1RAcP^‐/‐^ mice were sublingually infected with *C. albicans* (AHY940). RNA was extracted from tongue and gene expression determined by RT-qPCR. Results were quantified by ΔΔ*Ct* analysis compared against a naïve sample. Graph displays cumulative data from at least 3 independent experiments (n = 12 for each group). Differences between control and IL-1RAcP^‐/‐^ were analysed by Kruskal-Wallis test with Dunn’s post-test. *Csf3* *P = 0.0084. Data presented as median with interquartile range. (**b**) CD45^+^ and CD45^+^ Ly6G^+^ CD11b^+^ cells were quantified from at least 3 independent experiments (n = 3 for each group). Differences between control and IL-1RAcP^‐/‐^ were analysed by 2-way ANOVA with Bonferroni’s post-test. Data presented as mean ± s.e.m. (**c-g**) Control and IL-1RAcP^‐/‐^ mice were sublingually infected with *C. albicans* (AHY940). (**c**) Gating strategy for isolating CD45^+^ Ly6G^+^ CD11b^+^ cells completed on day 2 post-OPC control sample. (**d-f**) Bone marrow was harvested from control and IL-1RAcP^‐/‐^ femurs, single cells isolated and stained with anti-CD45, anti-Ly6G, anti-Ly6C, and anti-CD11b antibodies and/or their appropriate isotypes before being analysed by flow cytometry. CD45^+^ CD11b^+^ Ly6G^HI^ Ly6C^INT^ cells were identified as mature neutrophils. (**d**) Flow plots representative of data from at least 3 independent experiments, gating shown as single, CD45^+^, CD11b^+^ and as percentage (%) of total CD45^+^ CD11b^+^ cells. (**e**) CD45^+^ CD11b^+^ Ly6G^HI^ Ly6C^INT^ cells were quantified from at least 3 independent experiments (n = 3 for each group). Differences between control and IL-1RAcP^‐/‐^ at each timepoint were analysed by 2-way ANOVA with Bonferroni’s post-test. *adj.*P* = 0.0439, **adj.*P* = 0.0023. Data presented as mean ± s.e.m. (**f**) Gating strategy for isolating CD45^+^ CD11b^+^ Ly6G^HI^ Ly6C^INT^ cells completed on day 2 post-OPC control sample. (**g**) CD101 expression was examined on CD45^+^ Ly6G^+^ cells isolated from tongue following OPC. CD101 expression displayed as histogram. Isotype control set from IL-1RAcP^‐/‐^ day 2 sample. CD101 expression shown from first significant infiltration of neutrophils (day 1 control and day 2 IL-1RAcP^‐/‐^). Flow plots and histograms are representative of 3 independent experiments. Statistical significance: adjusted (adj.), *P < 0.05, **P < 0.01. Source data

Neutrophil recruitment is a defining feature of OPC and we observed high quantities of inflammatory infiltrate in IL-1RAcP^−/−^ mice 2 days after OPC (Fig. 1f). To investigate neutrophil recruitment, we induced OPC in IL-1RAcP^‐/‐^ and control mice, collected tongue tissue and stained histological sections with haematoxylin and eosin (H&E). Interestingly, extensive neutrophil infiltration was observed only in IL-1RAcP^‐/‐^ at day 2 (Fig. 3a). Next, to explore how the IL-1 family influences the recruitment of neutrophils to the mucosa, we quantified the expression of *Cxcl1* and *Csf3* (key neutrophil recruiting chemokines). The rapid induction (day 1) of both *Cxcl1* and *Csf3* was highly dependent on the IL-1 family; however, by day 2 both genes were potently induced in the IL-1RAcP^‐/‐^ model (Fig. 3b and Extended Data Fig. 3a). Strikingly, using flow cytometry, neutrophil recruitment in IL-1RAcP^‐/‐^ mice was ~8-fold lower on day 1. However, by day 2, recruitment surged dramatically, with neutrophil numbers increasing ~40-fold compared with day 1 and exceeding those in control mice by ~12 fold (Fig. 3c–e and Extended Data Fig. 3b,c). To further explore this neutrophil infiltration, we examined emergency granulopoiesis by assessing changes in bone marrow composition (Extended Data Fig. 3d–f). Although a modest release of mature neutrophils was observed in control mice, we identified a large but delayed release of neutrophils in IL-1RAcP^‐/‐^ mice at day 2. In concordance, while mature neutrophils were recruited into the oral mucosa in the control mice at day 1, the substantial day 2 infiltration of neutrophils in the IL-1RAcP^‐/‐^ model contained both mature and immature neutrophils (Extended Data Fig. 3g). Our data demonstrate that the early recruitment of neutrophils during OPC is dependent on IL-1 family signalling, but once OPC becomes severe IL-1 family independent mechanisms provide redundancy.

### Epithelial IL-1 family signalling promotes the timely resolution of mucosal infection

Having evaluated the importance of the IL-1 family during mucosal fungal infection, we next sought to identify the cellular sources of IL-1 family signalling and define how this signalling elicited protective immunity. In addition, we hypothesized that the substantial recruitment of neutrophils recovered host protection in the IL-1RAcP^‐/‐^ model; however, the mechanisms underlying this were unclear.

**Fig. 4:**
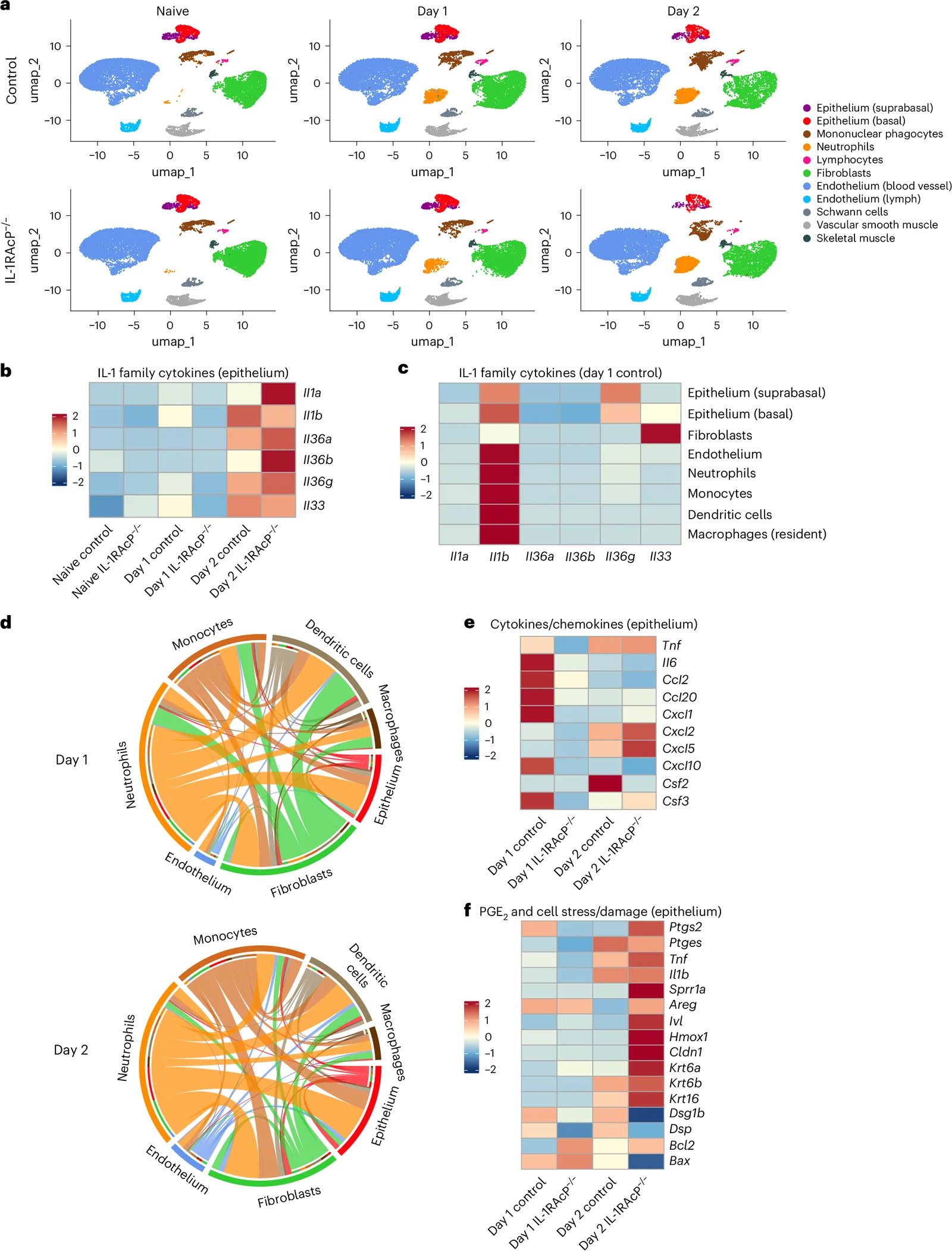
Epithelial IL-1 family signalling promotes the timely resolution of mucosal infection. Control and IL-1RAcP^‐/‐^ mice were sublingually infected with *C. albicans* (AHY940). Tongue tissue was collected and processed into single-cell suspensions for scRNA-seq (two tongues pooled per sample, ~20,000 cells collected per sample). Data were analysed using Seurat and CellChat. **a**, UMAP of clustered cell populations present in the oral mucosa of control and IL-1RAcP^‐/‐^ mice at baseline and at day 1 and day 2 post-OPC. Clusters are annotated by cell identity as shown in the accompanying legend. **b**,**c**, Heat map data displayed as scaled *z* scores; analyses represent qualitative assessment per sample. Heat map showing average expression of IL-1 family cytokines in the oral epithelium (basal and suprabasal combined) across the OPC time course (naive, day 1, day 2) in control and IL-1RAcP^‐/‐^ mice. Data are displayed as scaled *z* scores indicating highest relative expression of each cytokine across genotypes and timepoints (**b**). Heat map of average IL-1 family cytokine expression across oral mucosal cell types at day 1 of OPC in control mice, shown as scaled *z* scores indicating the cytokine most highly expressed in each population (**c**). **d**, CellChat analysis of IL-1 family cytokine–receptor interaction networks in the oral mucosa at day 1 and day 2 of OPC from control mice, scaled for population size. Thicker arrows infer higher communication and arrows point from sender to receiver in the colour of the sending cluster; small half-circles on outgoing communications indicate target clusters. **e**,**f**, Heat map data displayed as scaled *z* scores; analyses represent qualitative assessment per sample. Heat map displaying average chemokine expression in the oral epithelium (basal and suprabasal combined) across the OPC time course (day 1, day 2) in control and IL-1RAcP^‐/‐^ mice. *Z* scores indicate highest expression of each chemokine across timepoints and genotypes (**e**). Heat map showing average expression of PGE_2_-producing enzymes and epithelial damage/stress markers in the oral epithelium across the OPC time course (day 1, day 2) in control and IL-1RAcP^‐/‐^mice (**f**).

**Extended Data Fig. 4:**
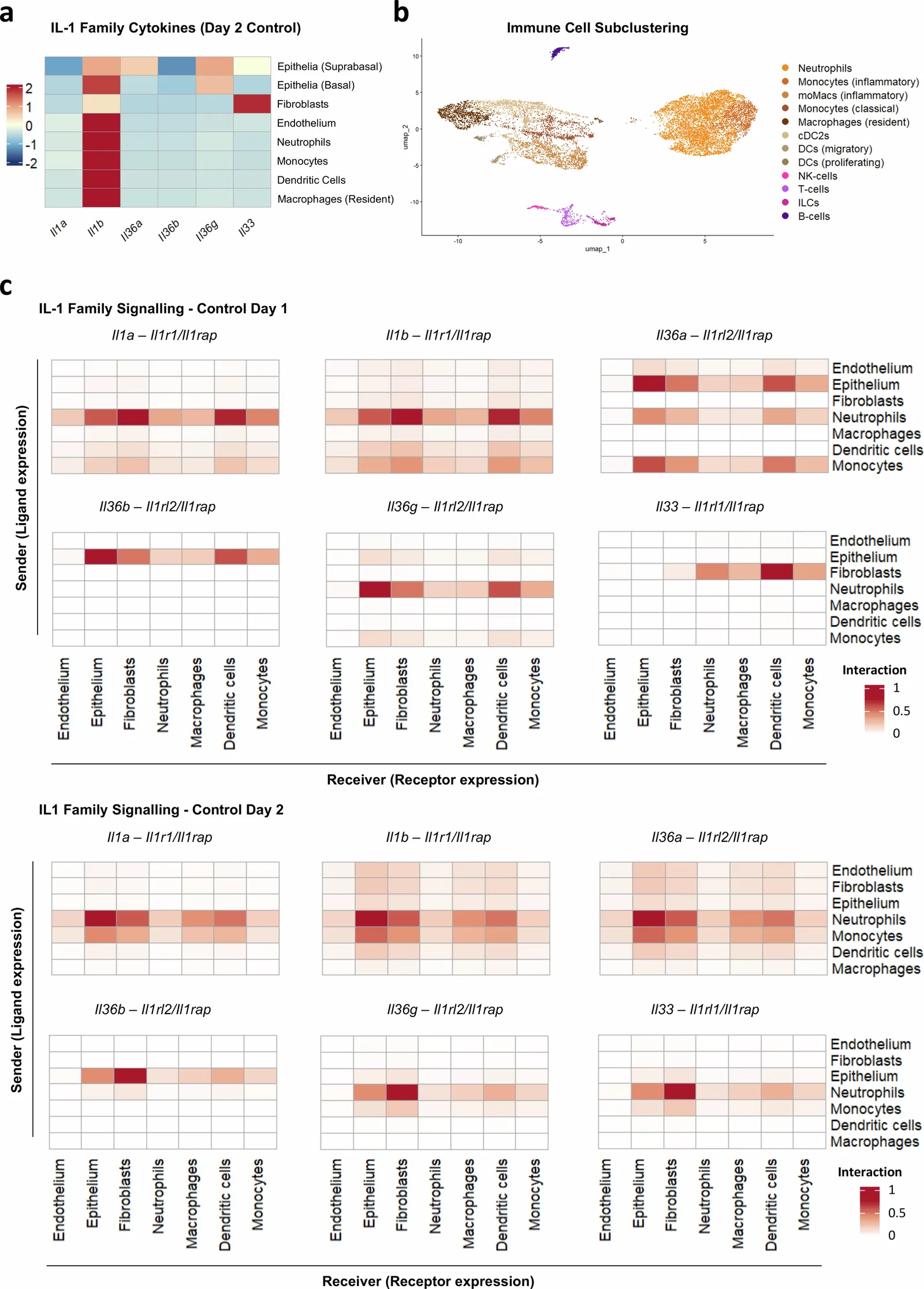
Single-cell analysis identifies cellular sources and targets of IL-1 family signalling during OPC. (**a-c**) Control and IL-1RAcP^‐/‐^ mice were sublingually infected with *C. albicans* (AHY940). Tongue tissue was collected and processed into single-cell suspensions for scRNA-seq (2 tongues pooled per sample, ~20,000 cells per sample). Data were analysed using Seurat and CellChat. (**a**) Heatmap showing average expression of IL-1 family cytokines across cell types present in the oral mucosa at day 2 of OPC in control mice. Data are displayed as scaled *z*-scores indicating the cytokine most highly expressed in each cell type. (**b**) UMAP of myeloid (*Ptprc*-expressing) populations subclustered from the oral mucosa of control and IL-1RAcP^‐/‐^ mice at naïve, day 1, and day 2 of OPC (combined). Individual subclusters represent distinct immune cell types as described in displayed legend. (**c**) Heatmap of IL-1 family sender–receiver communication pairings in the oral mucosa at day 1 and day 2 of OPC in control mice. Sender clusters (y-axis) were probed for IL-1 family ligand expression, while receiver clusters (x-axis) were assessed for co-expression of cognate receptors and IL-1RAcP. Colour scale represents relative communication intensity across cluster pairs and analyses represent qualitative assessment.

Thus, we induced OPC in IL-1RAcP^‐/‐^ and control mice, collected tongue tissue and undertook single-cell RNA sequencing (scRNA-seq) at day 1 and day 2 post-infection. Seurat UMAP clustering revealed stable populations of epithelia, endothelia, fibroblasts, Schwann cells and muscle cells across samples, together with variable populations of lymphocytes, neutrophils and mononuclear phagocytes (Fig. 4a). To determine the initial source of IL-1 family signals during OPC we examined IL-1 family expression in epithelia. In agreement with our RNA-sequencing results (Fig. 1a), epithelial cells expressed *Il1a*, *Il1b*, *Il33* and *Il36g* with prompt expression dependent on IL-1 family signalling. By day 2, epithelia additionally expressed *Il36a* and *Il36b* (Fig. 4b). While we hypothesized that epithelia would serve as the initial source of IL-1 family signals due to their proximity to the invading pathogen, we identified substantial *Il1b* expression from resident cells and infiltrating myeloid cells, and *Il33* expression from fibroblasts (Fig. 4c and Extended Data Fig. 4a,b).

**Extended Data Fig. 5:**
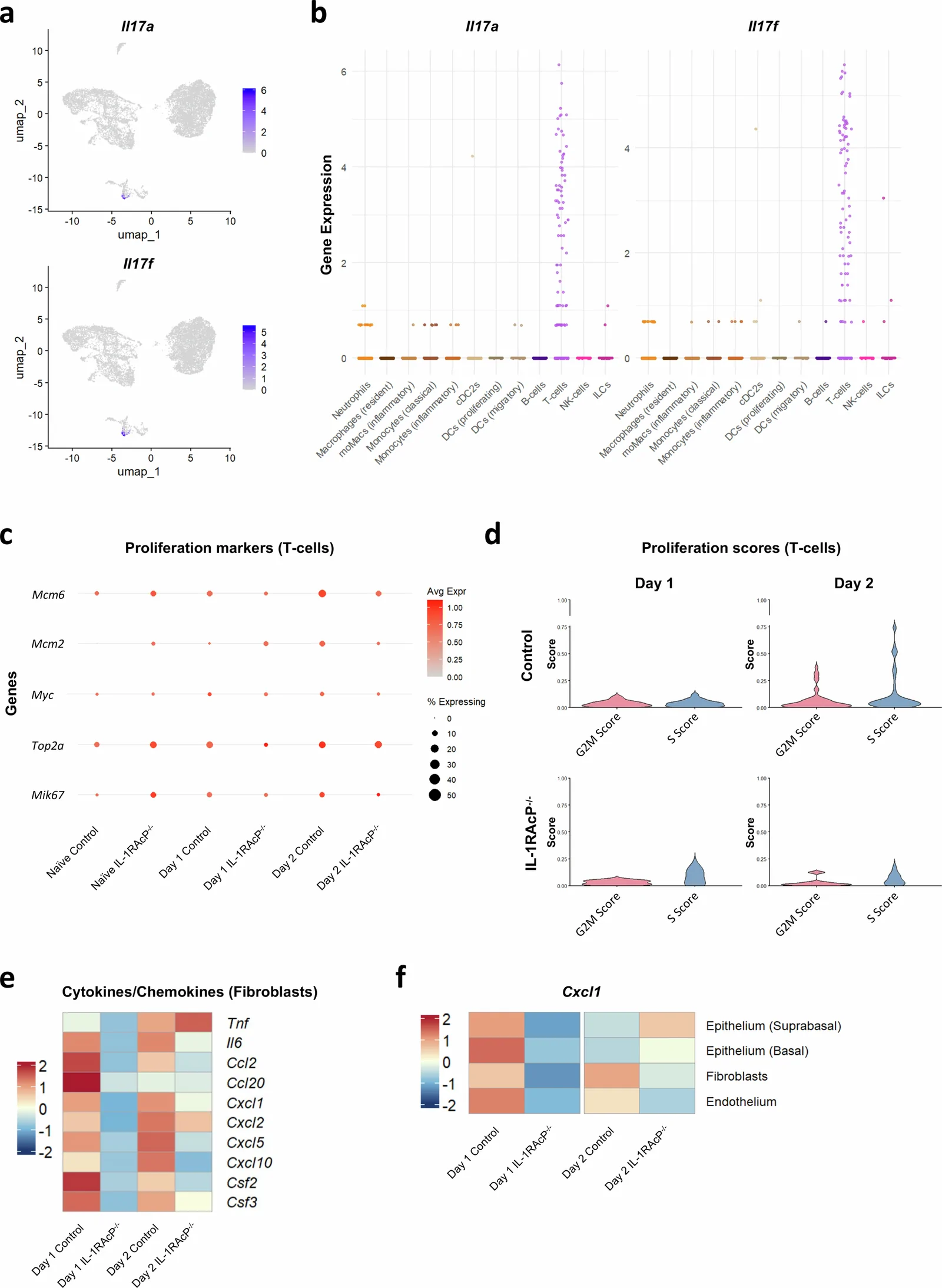
Single-cell analysis reveals T-cell IL-17 responses and chemokine expression during OPC. (**a-f**) Control and IL-1RAcP^‐/‐^ mice were sublingually infected with *C. albicans* (AHY940). Tongue tissue was processed into single-cell suspensions for scRNA-seq (2 tongues pooled per sample, ~20,000 cells per sample). Data were analysed using Seurat. (**a-b**) Expression of IL-17 family cytokines localized to the T-cell cluster. (**a**) Feature plot showing *Il17a/f* expression across immune clusters. (**b**) Scatter plot illustrating *Il17a/f* expression levels in immune cluster. (**c**) DotPlot highlighting the proportion of cells expressing (dot size) and average expression level (colour intensity) of proliferative signature genes in T-cells across the OPC time course (naïve, day 1, day 2) in control and IL-1RAcP^‐/‐^ mice. (**d**) Violin plot showing T-cell S-phase and G2M-phase scores computed with *CellCycleScoring* (Seurat) across the OPC time course (day 1, day 2) in control and IL-1RAcP^‐/‐^ mice. (**e**) Heatmap of average chemokine expression in fibroblasts across the OPC time course (naïve, day 1, day 2) for control and IL-1RAcP^‐/‐^ mice. Data are displayed as scaled *z*-scores indicating highest expression of each chemokine across timepoints and genotypes. (**f**) Heatmap showing average *Cxcl1* expression in non-immune cells across the OPC time course (day 1, day 2) in control and IL-1RAcP^‐/‐^ mice, presented as scaled *z*-scores indicating maximal *Cxcl1* expression across genotypes and timepoints.

Having identified the cellular sources of IL-1 family ligands, we next assessed IL-1 family communication using CellChat analysis. This revealed extensive IL-1 family signalling between epithelial cells and fibroblasts, highlighting interactions involving IL-1α, IL-1β, IL-36α, IL-36β and IL-36γ, and suggesting that this axis functions as a primary signalling hub. We also identified potential myeloid cell IL-1 family signalling with neutrophils, dendritic cells and monocyte receptor expression consistent with responsiveness to IL-1α, IL-1β, IL-36α, IL-36γ and IL-33 (Fig. 4d and Extended Data Fig. 4c). We next examined *Il17a/f* expression and localized this to T cells (Extended Data Fig. 5a,b). Interestingly, T cells appeared to infiltrate into the infection site before a small population may have started proliferating (Extended Data Fig. 5c,d).

After establishing epithelia and fibroblasts as the primary recipients of IL-1 family signalling, we investigated how IL-1 family signalling shaped the expression of key cytokines and chemokines in these populations. Strikingly, in control mice, the timely expression of *Cxcl1*, *Csf2*, *Csf3*, *Cxcl10*, *Ccl2*, *Ccl20* and *Il6* (responsible for recruiting neutrophils, monocytes and T cells) was highly dependent on IL-1 family signalling. By contrast, these responses were largely absent in IL-1RAcP^‐/‐^ mice, which exhibited delayed epithelial expression of *Cxcl1*, and potent epithelial *Cxcl2* and *Cxcl5* expression, probably responsible for the eventual infiltration of neutrophils (Fig. 4e and Extended Data Fig. 5e,f).

We next investigated which IL-1 family-independent upstream pathways could initiate the recruitment of neutrophils. Inducing OPC in IL-1RAcP^‐/‐^ mice resulted in extensive fungal invasion (Fig. 1f) and a delayed but substantial IL-17 response (Fig. 2b and Extended Data Fig. 2b), suggesting that damage-associated pathways may drive IL-17 and subsequent epithelial chemokine production. Cyclooxygenase-2 (COX-2, *Ptgs2*), which generates the bioactive lipid Prostaglandin E_2_ (PGE_2_), can be induced both by IL-1 family cytokines^20,21^ and by cellular stress/damage^22,23^. Importantly, PGE_2_ can act synergistically with IL-1 to enhance IL-17 expression^24^, but also potently amplifies IL-17 production from γδ^+^ cells independently of IL-1 signalling^25^. Therefore, we assessed whether this pathway was induced in our model (Fig. 4f). While *Ptgs2* and *Ptges* were induced in control mice, we also identified their robust induction in IL-1RAcP^‐/‐^ mice at day 2. Importantly, the IL-1 family-independent induction of PGE_2_ was accompanied by the differential expression of several genes associated with barrier breakdown (*Cldn1*, *Dsg1b* and *Dsp*)^26,27^, wound healing (*Krt16*, *Krt6a* and *Krt6b*)^28^, cellular stress and dysfunction (*Sprr1a*, *Areg*, *Ivl* and *Hmox1*)^29–32^, and anti-apoptosis pro-survival signalling (*Bcl2*/*Bax*)^33^.

Our data demonstrate that epithelial cells are the initial source of IL-1 family cytokines, that rapid IL-1 family expression is itself dependent on IL-1 family signalling and that this response is subsequently amplified by the myeloid compartment. Functionally, IL-1 family signalling leads to the rapid epithelial and fibroblast-driven recruitment of neutrophils, monocytes and T cells. In the absence of IL-1 family signalling, immune cell recruitment is lost and infection-driven cellular damage and stress promote PGE_2_ pathway activation, which probably provides the redundancy that drives the delayed recruitment of neutrophils.

### Mucosal–systemic dissemination is governed by the IL-1 family and neutrophils

**Fig. 5:**
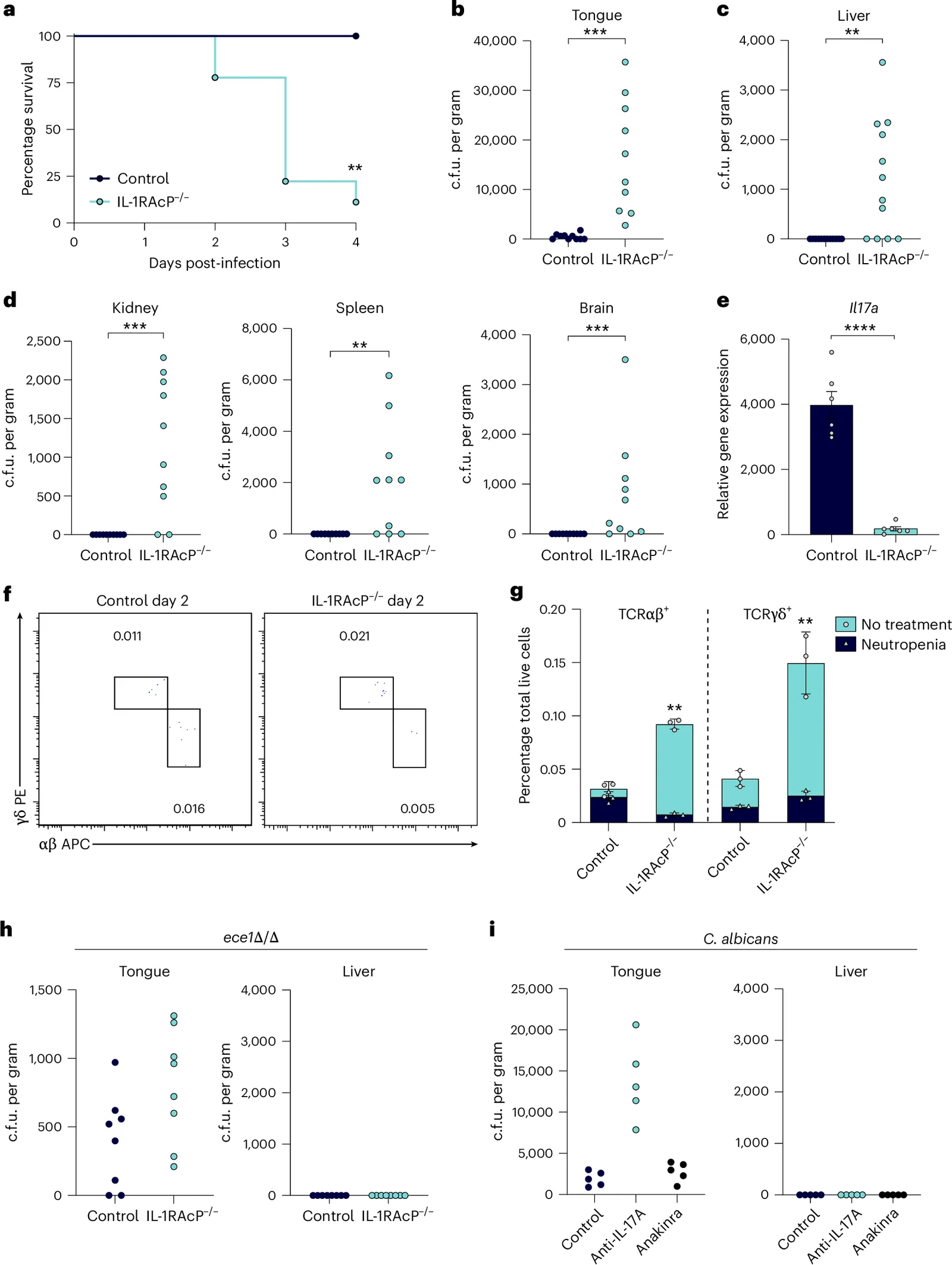
The IL-1 family and neutrophils restrict invasion and restrain *C. albicans* within the mucosa. Neutrophils were depleted in control and IL-1RAcP^‐/‐^ mice by intraperitoneal administration of anti-Ly6G antibody starting 1 day before sublingual administration of *C. albicans* (AHY940). **a**, Mice were weighed and scored daily and any mice exceeding experimental severity limits were culled. Graph displays cumulative data from at least three independent experiments (*n* = 12 for each group). Survival differences between control and IL-1RAcP^‐/‐^ were analysed by log-rank test. **P = 0.0067. **b**, c.f.u. was enumerated from tongue homogenate at day 4 (*n* = 10 for each group). **c**, c.f.u. was enumerated from liver homogenate at day 3 (*n* = 12 for each group). **d**, c.f.u. was enumerated from kidney, spleen and brain homogenate at day 4 (n = 10 for each group). In **b**–**d** each symbol represents one mouse. c.f.u. differences were analysed by Kruskal–Wallis test with Dunn’s post-test. Tongue ***P = 0.0003, liver **P = 0.0013, kidney ***P = 0.0007, spleen **P = 0.0031, brain ***P = 0.0007. **e**, RNA was extracted from tongue on day 2 and gene expression determined by RT–qPCR. Results were quantified by ΔΔ*Ct* analysis compared against a naive sample. Graph displays cumulative data from at least three independent experiments (*n* = 6 for each group). Differences between control and IL-1RAcP^‐/‐^ were analysed by Kruskal–Wallis test with Dunn’s post-test. ****P < 0.0001. Data presented as median with interquartile range. **f**, Tongue tissue was homogenized to single cells and stained with a live/dead dye, anti-CD45, anti-CD4, anti-TCRαβ and anti-TCRγδ antibodies and/or their appropriate isotypes before being analysed by flow cytometry. Flow plots representative of data from at least three independent experiment and gating shown as live (dead^+^ exclusion), single, CD45^+^, CD4^+^ and as percentage of total live cells. **g**, CD4^+^ αβ^+^ and CD4^+^ γδ^+^ cells were quantified from at least three independent experiments (*n* = 3 for each group) and differences between neutropenic and non-neutropenic for control and IL-1RAcP^‐/‐^ mice were analysed by two-way ANOVA with Bonferroni’s post-test. TCRαβ^+^ **adj. *P* = 0.0013, TCRγδ^+^ **adj. *P* = 0.0045. Data are presented as mean ± s.e.m. **h**, Neutrophils were depleted in control and IL-1RAcP^‐/‐^ mice by intraperitoneal administration of anti-Ly6G antibody starting 1 day before the sublingual administration of *C. albicans ece1*Δ/Δ. c.f.u. was enumerated from tongue and liver homogenate at day 4. Each symbol represents one mouse (*n* = 8 for each group). c.f.u. differences were analysed by Kruskal–Wallis test with Dunn’s post-test. **i**, Neutrophils were depleted in control mice by intraperitoneal administration of anti-Ly6G antibody starting 1 day before sublingual administration of *C. albicans* (AHY940). Anti-IL-17A antibody or Anakinra was administered by intraperitoneal administration starting 1 day before sublingual administration of *C. albicans* (AHY940). c.f.u. was enumerated from tongue and liver homogenate at day 4. Each symbol represents one mouse (*n* = 5 for each group). c.f.u. differences were analysed by Kruskal–Wallis test with Dunn’s post-test. Statistical significance: adj. *P < 0.05, **P < 0.01, ***P < 0.001, ****P < 0.0001. Source data

**Extended Data Fig. 6:**
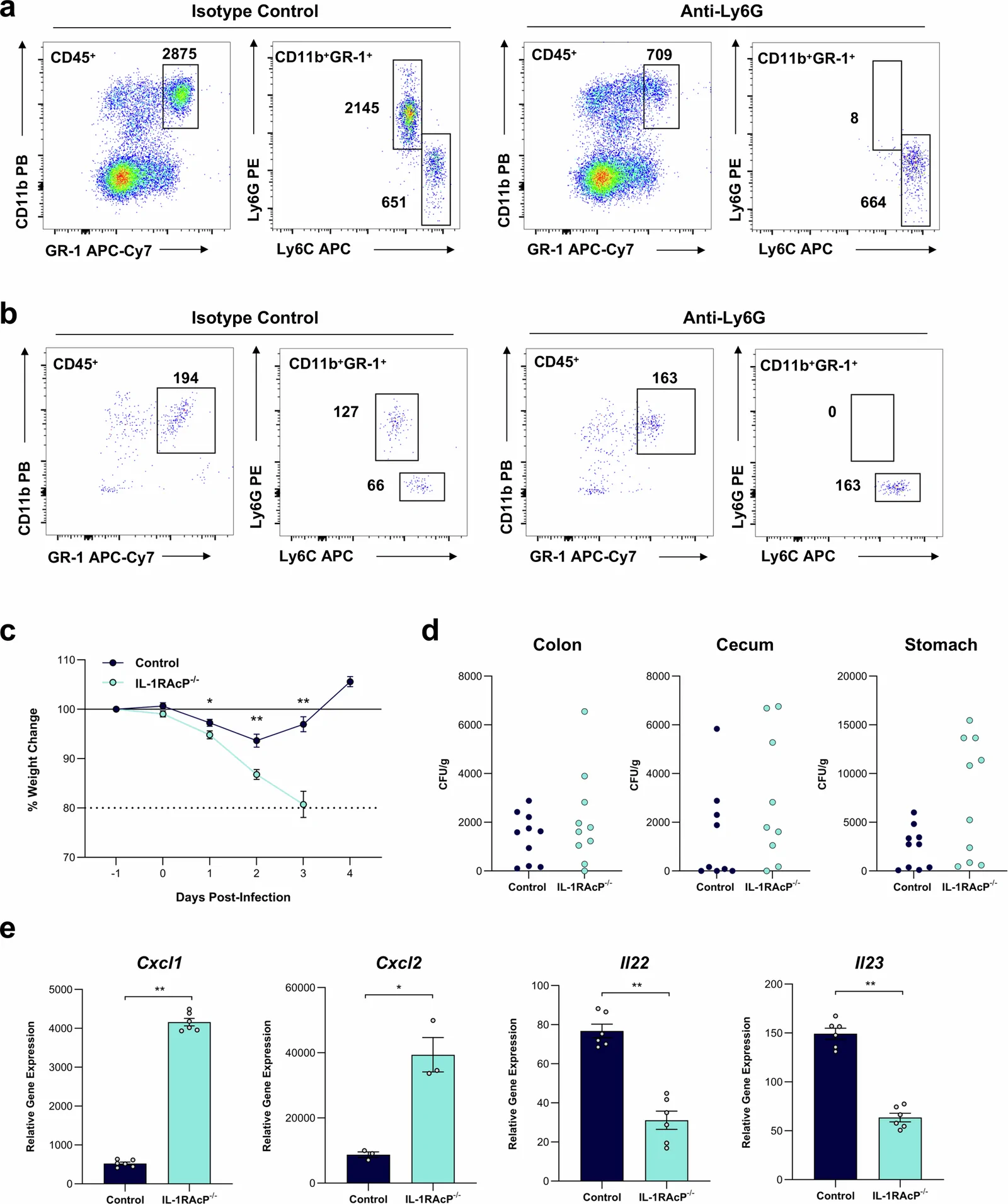
Combined neutrophil depletion and IL-1 family deficiency result in profound susceptibility to mucosal C. albicans infection. (**a**) Blood was drawn from control mice 1 day after intraperitoneal administration of anti-Ly6G antibody or isotype control and stained with anti-CD45, anti-CD11b, anti-GR-1, anti-Ly6G, and anti-Ly6C antibodies and/or their appropriate isotypes before being analysed by flow cytometry. CD45^+^ GR-1^+^ CD11b^+^ cells were gated forwards. Ly6G^+^ Ly6C^INT^-cells were identified as neutrophils and Ly6G^−^ Ly6C^HI^ cells were identified as monocytes. Flow plots representative of data from 2 independent experiments, quantification represents absolute numbers of cells. (**b-e**) Neutrophils were depleted in control and IL-1RAcP^‐/‐^ mice by intraperitoneal administration of anti-Ly6G antibody starting 1 day prior to sublingual administration of *C. albicans* (AHY940). (**b**) After 1 day tongue tissue was homogenised to single cells and stained with a live/dead dye, anti-CD45, anti-CD11b, anti-GR-1, anti-Ly6G, and anti-Ly6C antibodies and/or their appropriate isotypes before being analysed by flow cytometry. CD45^+^ GR-1^+^ CD11b^+^ cells were gated forwards. Ly6G^+^ Ly6C^INT^-cells were identified as neutrophils and Ly6G^−^ Ly6C^HI^ cells were identified as monocytes. Flow plots representative of data from 2 independent experiments, quantification represents absolute numbers of cells. (**c**) Weight change of grouped control and IL-1RAcP^‐/‐^ mice data from at least 3 independent experiments (n = 12 for each group). Each timepoint between groups was compared by2-way ANOVA with Bonferroni’s post-test. Day 1 *adj.*P* = 0.0432, Day 2 **adj.*P* = 0.0091, Day 3 **adj.*P* = 0.0074. Data presented as mean ± s.e.m. Dotted line represents experimental limit. (**d**) CFU was enumerated from colon, cecum and stomach homogenate at day 4. Each symbol represents one mouse (n = 10 per group for colon and stomach, n = 9 per group for cecum). (**e**) RNA was extracted from tongue and gene expression determined by RT-qPCR. Results were quantified by *ΔΔCt* analysis compared against a naïve sample. Graph displays cumulative data from at least 3 independent experiments (n = 6 for *Cxcl1*, *Il22* and *Il23*; n = 3 for *Cxcl2*). Differences between control and IL-1RAcP^‐/‐^ were analysed by Kruskal-Wallis test with Dunn’s post-test. *Cxcl1* **P = 0.001, *Cxcl2* *P = 0.025, *Il22* **P = 0.0022, *Il23* **P = 0.0022. Data presented as median with interquartile range. Statistical significance: adjusted (adj.), *P < 0.05, **P < 0.01. Source data

Our data demonstrate that IL-1 family signalling is critical for the initial control of OPC infection (day 1), but that even without IL-1 family signalling neutrophils are eventually recruited (day 2) and infection is resolved. We therefore hypothesized that IL-1 family signalling and neutrophils cooperate to provide cumulative protection against OPC as the infection progresses. To test this, we depleted neutrophils (Extended Data Fig. 6a,b) in both IL-1RAcP^‐/‐^ and control mice using anti-Ly6G antibodies and induced OPC. Strikingly, we observed severe mucosal infection in neutropenic IL-1RAcP^‐/‐^ mice, which succumbed to the infection by day 4, confirming that neutrophils provide potent redundancy (Fig. 5a and Extended Data Fig. 6c). By contrast, neutropenic (wild-type) control mice largely cleared the infection with minimal weight loss (Fig. 5b and Extended Data Fig. 6c). *C. albicans* could be recovered from the stomach, colon and caecum (via swallowing) in both neutropenic control and neutropenic IL-1RAcP^‐/‐^ mice at similar levels (Extended Data Fig. 6d). However, *C. albicans* could only be recovered from internal organs of neutropenic IL-1RAcP^‐/‐^ mice, with the fungus initially targeting the liver (day 3) but also spleen, kidney and brain (day 4) (Fig. 5c,d). Importantly, the liver was the predominant target of disseminated *C. albicans*, mimicking the primary organ *C. albicans* targets in severely immunocompromised patients^4,34,35^. Our data demonstrate that IL-1 family signalling and neutrophils cooperate to prevent fungal translocation across mucosal barriers and subsequent life-threatening systemic infections.

Since protection against *C. albicans* dissemination was provided by either the IL-1 family or neutrophils, we next investigated whether neutrophils were responsible for the delayed recruitment of TCRαβ^+^ and TCRγδ^+^ cells in IL-1RAcP^‐/‐^ mice at day 2 post infection. Notably, depletion of neutrophils blocked IL-17A expression and TCRαβ^+^ and TCRγδ^+^ infiltration (Fig. 5e–g). Interestingly, while the expression of the IL-17-associated genes *Il22* and *Il23* was also reduced, the expression of neutrophil attracting chemokines was significantly increased despite the absence of neutrophils (Extended Data Fig. 6e).

**Extended Data Fig. 7:**
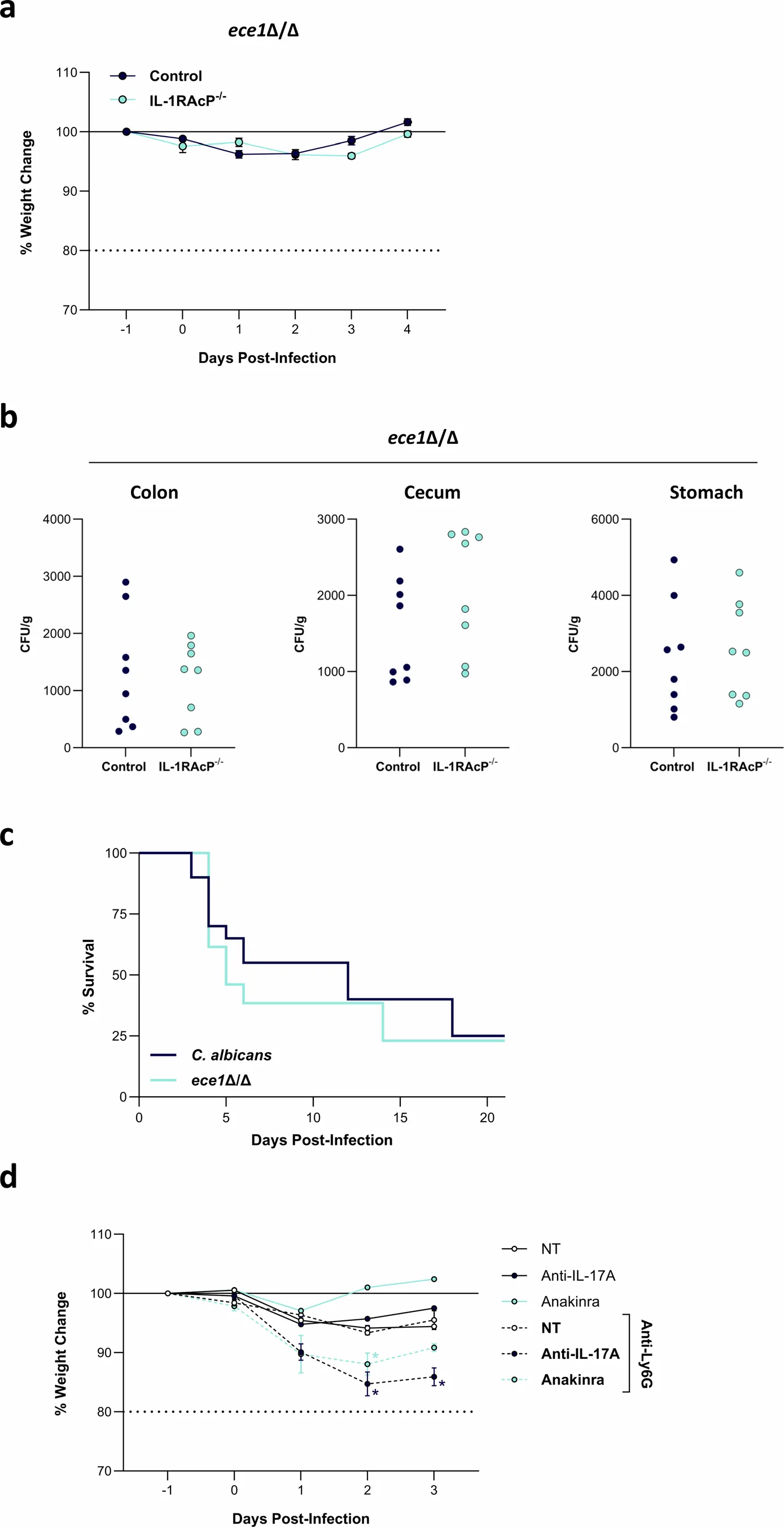
Candidalysin is required for mucosal dissemination. (**a-b**) Neutrophils were depleted in control and IL-1RAcP^‐/‐^ mice by intraperitoneal administration of anti-Ly6G antibody starting 1 day prior to the sublingual administration *of C. albicans ece1*Δ/Δ. (**a**) Weight change of grouped control and IL-1RAcP^‐/‐^ mice data from 2 independent experiments (n = 8 for each group). Each timepoint was compared to the day 0 value by 2-way ANOVA with Bonferroni’s post-test. Data presented as mean ± s.e.m. (**b**) CFU was enumerated from colon, cecum and stomach homogenate at day 4. Each symbol represents one mouse (n = 8 for each group). (**c**) Control mice were intravenously challenged with *C. albicans* (AHY940) (n = 20) or *ece1*Δ/Δ (n = 13,) and monitored for 21 days. Survival differences were compared between the two genotypes using a Mantel-Cox log rank test. (**d**) Neutrophils were depleted in control mice by intraperitoneal administration of anti-Ly6G antibody starting 1 day prior to sublingual administration of *C. albicans* (AHY940). Anti-IL-17A antibody or Anakinra was administered by intraperitoneal administration starting 1 day prior to sublingual administration of *C. albicans* (AHY940). Each line represents grouped control and IL-1RAcP^‐/‐^ mice data from 2 independent experiments (n = 5 for each group). Each timepoint was compared to the day 0 value by 2-way ANOVA with Bonferroni’s post-test. Anti-Ly6G and Anakinra-treated Day 2 *adj.*P* = 0.0403, Anti-Ly6G and Anti-IL-17A-treated Day 2 *adj.*P* = 0.0378, Anti-Ly6G and Anti-IL-17A-treated Day 3 *adj.*P* = 0.0246. Black dotted line represents experimental limit. Data presented as mean ± s.e.m. Statistical significance: adjusted (adj.), *P < 0.05. Source data

Candidalysin is vital for *C. albicans* infection and the induction of mucosal immunity^5–7,36^. To determine whether candidalysin was required for fungal dissemination, we challenged neutropenic IL-1RAcP^‐/‐^ and control mice with wild-type *C. albicans* (AHY940) and *ece1*Δ/Δ. Despite both fungal strains being equally capable of colonizing mucosal tissues, *ece1*Δ/Δ was unable to translocate across mucosal barriers and was absent from internal organs, demonstrating that candidalysin is essential for fungal dissemination (Fig. 5h and Extended Data Fig. 7a,b). Importantly, once *C. albicans* has breached the mucosa, candidalysin appears less critical as control mice were similarly susceptible to intravenous challenge with wild-type *C. albicans* (AHY940) or *ece1*Δ/Δ (Extended Data Fig. 7c).

Finally, we investigated whether IL-1 family members together controlled dissemination or whether IL-1R deficiency or IL-17 deficiency alone permitted dissemination. Thus, we challenged neutropenic control mice with anakinra (IL-1R antagonist) or anti-IL-17A antibodies and infected with wild-type *C. albicans* (AHY940). Anakinra had little effect, with mice displaying low oral fungal burden, no dissemination and weight loss recovering from day 3. Similarly, while anti-IL-17A antibodies rendered neutropenic mice more susceptible to persistent infection and greater weight loss, no fungal dissemination was observed (Fig. 5i and Extended Data Fig. 7d). Together, our data demonstrates the IL-1 family cooperates with neutrophils to prevent *C. albicans* dissemination and life-threatening fungal disease.

## Discussion

Host mechanisms that control microbial overgrowth to prevent mucosal disease and limit dissemination are poorly understood. Fungal infections are a serious threat to global health, particularly as the number of immunocompromised patients is increasing^37^. With limited therapeutic options and increasing antifungal resistance, there is an urgent need to identify host factors capable of controlling mucosal infection and preventing dissemination. Our results identify a central role for the IL-1 family in mediating rapid and protective immunity against mucosal *C. albicans* challenge. Crucially, the IL-1 family can prevent mucosal *C. albicans* infection from disseminating in the absence of neutrophils. Similarly, without IL-1 family signalling, a delayed neutrophil response resolves mucosal *C. albicans* infection preventing dissemination. Critically, absence of IL-1 family signalling coupled with neutropenia permits *C. albicans* dissemination from the mucosa, first to the liver and then into multiple organs, mimicking disease experienced by severely immunocompromised patients. Our data identify the IL-1 family as a key host risk factor for life-threatening disseminated disease.

The activation of host mucosal immunity by *C. albicans* through candidalysin-induced cellular damage is well described^5,8,12,38^. In agreement, we confirmed that the induction of *IL1A*, *IL1B*, *IL33* and *IL36G* was candidalysin dependent. Previous studies have demonstrated that both haematopoietic and non-haematopoietic sources of IL-1 contribute to protective host immunity^6^. Our findings establish the epithelia as the initial source of IL-1 family ligands, with epithelial cells and fibroblasts the primary responders and infiltrating myeloid cells further amplifying IL-1 family signalling.

Modelling IL-1 family deficiency in vivo using IL-1RAcP^‐/‐^ mice revealed a high susceptibility to OPC infection with deep and widespread hyphal invasion, which typically requires extensive immune suppression to reach such severity^39^. OPC infection was driven by candidalysin as *C. albicans* deficient in candidalysin (*ece1*Δ/Δ) was unable to establish OPC even in IL-1RAcP^‐/‐^ mice. Although clear and important antifungal roles for CLRs and TLRs have been described^40,41^, we observed no epithelial gene induction of either receptor family, corroborating their lack of involvement in driving rapid oral mucosal immunity during OPC^6,42^.

The barrier immune response against mucosal pathogens, including *C. albicans*, relies heavily on AMP production^43^. In patients, a defective AMP compartment leads to reduced candidacidal capacity and elevated *C. albicans* colonization^44^. While candidalysin promotes the induction of key AMPs including β-defensins and calprotectin^7^, the host drives this response through IL-1 family signalling. Interestingly, we found cathelicidin expression was independent of IL-1 family signalling, aligning with a previous study showing that cathelicidin expression does not require candidalysin^7^. IL-17 plays a pivotal role in AMP production by mediating the activation of IκBζ, which drives β-defensin 3 expression^17^. Critically, candidalysin was identified as a key driver of this signalling pathway. Thus, our data suggests that the IL-1 family is upstream of IL-17 in this pathway. As AMPs are critical for limiting the initial growth of *C. albicans*^45^, we propose that abolishing the IL-1 family-dependent AMP response permits *C. albicans* overgrowth.

In addition to AMPs, IL-17 expression is central to barrier protection. Defects in the IL-17 pathway enhance both mucosal and systemic *C. albicans* susceptibility in mice and humans^13,46,47^. IL-17 has been linked to neutrophil recruitment, with studies suggesting IL-17 assists neutrophil infiltration but recruitment is not IL-17 dependent^48,49^. IL-17 is predominantly produced in response to mucosal *C. albicans* infection by γδ T cells and innate-acting T_H_17 cells (characterized by their expression of TCRαβ) and requires IL-23 (ref. ^50^). Our results reveal that IL-1 family signalling drives the rapid recruitment of innate-acting T_H_17 cells and the early production of IL-17. Previous studies using IL-1R- and IL-36R-deficient models indicated both pathways independently but only partially control IL-17 production^6,12^. It was thought that innate-acting T_H_17 cells alone expand during acute OPC infection^6^; however, we observed the expansion of γδ T cells in the IL-1RAcP^‐/‐^ model. This suggests that γδ T cells might restore IL-17 expression through an alternative, IL-1–independent pathway. PGE_2_ can promote IL-17 expression by γδ T cells independently of IL-1 signalling^25^, and PGE_2_ can be induced through cellular stress or damage without IL-1 family signalling^22,23^. Thus, our results indicate that the severity of mucosal infection in IL-1RAcP^‐/‐^ mice induces PGE_2_ production, which may in turn restore IL-17 expression via γδ T cells. While γδ T cells are known to infiltrate into other tissues during disease^51^, even during peritoneal candidiasis^52^, their role in mucosal *C. albicans* infection requires further investigation.

Neutrophils comprise a key arm of the mucosal immune response and when recruited provide potent mucosal protection^53^. However, neutrophils are not universally protective. In vulvovaginal candidiasis and inflammatory bowel disease, candidalysin-induced IL-1β drives neutrophil recruitment and pathogenic inflammation^11^. Notably, blockade of IL-1R signalling with anakinra dramatically reduced neutrophil infiltration and disease severity in these settings^54,55^.

Our findings reinforce the vital protective role of neutrophils during OPC and demonstrate that their rapid recruitment requires IL-1R signalling^56^. Mechanistically, IL-1 family signalling promoted epithelial and fibroblast *Cxcl1* expression in control mice, whereas epithelial *Cxcl2* and *Cxcl5* were predominantly expressed in IL-1RAcP^‐/‐^ mice. *Cxcl1* can be amplified through IL-1 family-dependent feedback loops^54,55^, while *Cxcl1*, *Cxcl2* and *Cxcl5* are induced downstream of IL-17 signalling^56^. This suggests early IL-1 family feedback loops together with IL-17 promote timely neutrophil recruitment in wild-type mice. By contrast, IL-1RAcP^‐/‐^ mice may require PGE_2_-driven IL-17 induction to recruit neutrophils. As such, the role of PGE_2_ in mucosal *C. albicans* infection requires further investigation.

Although delayed, neutrophil recruitment in the absence of IL-1 family signalling was potent and included immature cells, indicating that emergency granulopoiesis can be initiated independently to counter severe OPC infection. The highly protective role of neutrophils during OPC raises the possibility that IL-1R blockade might exacerbate disease. However, anakinra treatment had limited impact during OPC, probably reflecting compensatory activity from other IL-1 family members and independent pathways. Taken together, our data suggest the IL-1 family plays a critical role in the mucosa controlling the release of AMPs, early IL-17 expression and rapid neutrophil recruitment. IL-1RAcP^‐/‐^ mice were highly susceptible to OPC but infection was eventually resolved through the IL-1 family-independent recovery of IL-17 expression and extensive infiltration of neutrophils.

Neutrophils are potent antifungal immune cells, particularly owing to their ability to kill large pathogens including *C. albicans* hyphae^57^, and neutropenia is the most widely accepted patient risk factor for invasive fungal disease^58^. Given this, we hypothesized neutrophils were resolving the severe infection in the IL-1RAcP^‐/‐^ model. In agreement, inducing neutropenia in IL-1RAcP^‐/‐^ mice resulted in a severe OPC infection and mortality within 4 days. Remarkably, we found that *C. albicans* translocated across mucosal barriers to invade the liver (day 3) and subsequently the spleen, kidney and brain (day 4), mimicking the dissemination of *C. albicans* to multiple organs in human disease^4,34^. This is particularly noteworthy as modelling dissemination in vivo has proven challenging. Current systemic *C. albicans* infection models, which are initiated via intravenous injection and lead to kidney infection, are useful for modelling catheter-related disease^59^, but fail to capture the critical events governing mucosal dissemination.

We found that inducing neutropenia in IL-1RAcP^‐/‐^ mice eliminated the ability to recover IL-17 expression or to recruit γδ T cells and innate-acting T_H_17 cells. Neutrophils can produce IL-17 (refs. ^60,61^), suggesting that infiltrating neutrophils may facilitate crosstalk with the IL-17 axis. While this interaction has been previously described^62^, it requires further investigation during OPC. Also, although IL-17 deficiency can result in chronic mucosal infection^63^ and enhances susceptibility to systemic infection^46,64^, we corroborate that blocking IL-17 in neutropenic wild-type mice does not result in dissemination^13^. This suggests that combinatorial IL-1 family signalling, rather than IL-17 alone, is the key immune factor restricting fungal dissemination.

In summary, this study reveals that deficiencies in IL-1 family signalling permit uncontrolled mucosal *C. albicans* infection that, when coupled with neutropenia, results in mucosal–systemic dissemination and mortality. While antibiotics, chemotherapeutic agents and dextran sodium sulfate are typically used to promote the dissemination of microbes^65^, we identify the IL-1 family as a host factor critical for the control of mucosal–systemic dissemination of an invasive fungal pathogen. Notably, patients with polymorphisms leading to deficient IL-1 family signalling are more susceptible to invasive candidiasis^66^ and disseminated fungal infection^67^. Previous studies have retrospectively stratified patient cohorts according to their immune function and their risk of acquiring fungal disease, but this has not been undertaken for the IL-1 family^68–70^. Our findings suggest that combinatorial IL-1 family function plays a crucial role in dissemination risk, offering potential for a personalized therapeutic approach. Consequently, therapeutically enhancing IL-1 family function to augment mucosal immunity and reduce dissemination could have substantial clinical implications.

## Methods

### *C. albicans* strains and culture

BWP17 + CIp30 (wild-type), *ece1*Δ/Δ (*ECE1* deficient) and *ece1*Δ/Δ+*ECE1* (*ECE1* revertant), (all previously described^5^) were used for TR146 infection and RNA-sequencing analysis. All subsequent data from the study used AHY940 (SC5314 a/α *leu2*Δ/*LEU2*) or *ece1*Δ/Δ (*leu*2Δ/*LEU2 ece1*ΔΔ), an *ECE1* ORF deletion mutant constructed using CRISPR–Cas9 in parental strain AHY940 (refs. ^71,72^). All *C. albicans* strains were plated on yeast extract peptone dextrose (YPD) agar, cultured for 16–20 h in YPD broth, washed three times with PBS and resuspended at the required concentration in PBS.

### TR146 cell culture, treatment and RNA sequencing

TR146 human buccal epithelial squamous cell carcinoma cells (established from human female^73^) acquired from Cancer Tools (151425) and the European Collection of Authenticated Cell Cultures (10032305) were cultured in Dulbecco’s modified Eagle medium/nutrient mixture F12 (DMEM/F12) (Gibco, Thermo Fisher Scientific) supplemented with 10% (v/v) fetal bovine serum (FBS; Gibco, Thermo Fisher Scientific) and 1% penicillin–streptomycin (Gibco, Thermo Fisher Scientific) at 37 °C 5% CO_2_. Before treatment, confluent TR146 cells were serum-starved overnight in DMEM/F12 medium.

For RNA sequencing, TR146 cells were infected with *C. albicans* strains or PBS for 4 h at a multiplicity of infection of 10 (10 fungal cells to 1 epithelial cell), or 3 or 15 µM candidalysin in deionized water or a deionized water control for 6 h. Total RNA from TR146 monolayers was isolated using the Nucleospin II kit (Macherey-Nagel, Thermo Fisher Scientific). RNA quality control was performed with an Agilent RNA 600 Nano Kit using a Bioanalyzer. Libraries were prepared using a TruSeq RNA Sample Preparation kit V2 (Illumina). Paired-end raw sequencing data (FASTQ files) were aligned to human reference (GRCh38) from ensemble release 92 using ‘Kallisto’ software (v0.46.1)^74^ to quantify transcripts per million (TPM) and count values of the transcripts. The gene-level count and TPM values were calculated from the transcript-level abundance and counts using the R package ‘tximport’^75^. Differential expression analyses were performed based on gene-level counts (having Entrez ID and the median TPM >1 across all samples) using the R package ‘DESeq2’ and Wald test *P* value (FDR <0.01). The R package ‘org.Hs.eg.db’ was used for ID mapping and Hierarchical clustering with Ward. D2 and Euclidean distance were used to cluster the samples based on the similarity matrix. Additionally, a principal component analysis that visualized using R package ‘ggplot2’ was performed based on the log transformed TPMs. Volcano plots were generated with VolcaNoseR (^76^).

### In vivo murine studies

Mice were maintained and used according to King’s College London institutional and UK Home Office guidelines. This study was performed in strict accordance with the Project License (PP6711863). The animal care and use protocol adhered to the Animals (Scientific Procedures) Act 1986. B6;129S1-Il1raptm1Roml/J were purchased from Jax (RRID: IMSR_JAX:003284) and backcrossed for ten generations to C57BL/6J (RRID: IMSR_JAX:000664). Throughout the study IL-1RAcP^‐/‐^ represent the backcrossed B6;129S1-Il1raptm1Roml/J, and control mice represent C57BL/6J. Mice were housed at 20–23 °C ambient temperature, 45–55% humidity and with 12-h light/dark cycles. Male and female 8–12-week-old mice were used in the experiments.

### Murine OPC model

OPC was induced as previously described^39^. In brief, mice were anaesthetised for 75 min with an intraperitoneal injection of 75 mg kg^−1^ ketamine and 1 mg kg^−1^ domitor. A swab soaked in 10^7^ c.f.u. ml^−1^ *C. albicans* yeast in sterile PBS was placed sublingually for 75 min. After 1, 2, 3 or 4 days, mice were killed, the tongue excised and divided longitudinally in half. One half was weighed, homogenized using a Gentle MACS (Miltenyi Biotec) and serial dilutions were plated on YPD agar containing 50 μg ml^−1^ chloramphenicol. The plates were cultured at 37 °C for 24 h and c.f.u. was calculated per gram of organ. The other half was processed for RNA extraction, flow cytometry or histochemistry.

### Murine intravenous *C. albicans* infection model

Mice were challenged with 10^5 ^*C. albicans* yeast in sterile PBS intravenously administered into the tail vein. After 21 days, or once experimental limits were reached, mice were killed.

### Histology

Mouse tongue tissue was collected, formalin fixed, dehydrated and paraffin embedded before 5-µm sections were cut and mounted on slides. Slides then underwent periodic acid–Schiff staining or H&E staining following standard protocols. In brief for PAS staining, sections were treated with 1% periodic acid for 10 min followed by Schiff reagent for 10 min before counterstaining with Harris Hamotoxylin. In brief for H&E staining, sections were treated with Harris Hamotoxylin before washing with water and counterstaining with eosin.

### Gene expression analysis

Half tongue was collected into RNALater (Thermo Fisher Scientific) and homogenized using a Gentle MACS (Miltenyi Biotec). Total RNA was collected using an RNeasy Kit (Qiagen). cDNA was generated using the QuantiTect Rev. Transcription Kit (Qiagen). RT–qPCR was performed using a QuantiNova SYBR Green PCR kit (Qiagen) with predesigned primer pairs (Supplementary Data Table 1) purchased from Merck. RT–qPCR was run on a QuantStudio 7 (Thermo Fisher Scientific) and data collected using Quantstudio Real-Time PCR software (v1.7.2). Gene expression was calculated using ΔΔ*Ct* where the value of a control (typically naive) sample was set at 1.

### Tongue digestion and flow cytometry

Half tongue was collected on ice and homogenized using a Gentle MACS (Miltenyi Biotec) with a tumour dissociation kit (Miltenyi Biotec). Single-cell homogenate was passed through a 70-µm filter and washed with pre-cooled RPMI 1640 without phenol red (Gibco, Thermo Fisher) before being counted and resuspended in pre-cooled PBS. Cells were live/dead stained with Zombie Green Fixable Viability Kit (Biolegend) for 30 min at 4 °C and then washed in pre-cooled FACS Buffer (PBS 3% FBS 2 mM EDTA). Cells were blocked with TruStain FcX (anti-mouse CD16/32) (Biolegend) for 15 min at 4 °C before being stained with the required antibodies and/or isotypes (Supplementary Table 2) for 30 min at 4 °C. Cells were washed three times with pre-cooled FACS buffer before being run on a BD FACSCanto II (BD Biosciences). Data were collected using BD FACS DIVA (v8.0.1). Results were analysed using FlowJo software (v10.10.0) (BD Biosciences).

### scRNA-seq

Twelve mice in total were used for scRNA-seq analysis. All infected mice were inoculated with the same culture of *C. albicans* (AHY940) in one infection procedure at the same time.

Whole tongue was collected on ice and processed to single cells as described above using sorting buffer (PBS 0.5% fatty acid-free BSA (Sigma)) instead of FACS buffer. Single-cell homogenate isolated from two mice, one male and one female, for each genotype and timepoint, was pooled and sorted on a BD Aria 2 (BD Biosciences). Live cells were collected for scRNA-seq. Samples were kept at 4 °C throughout the sorting process. Before scRNA-seq analysis, sample viability of >80% was confirmed through acridine orange/propidium iodide staining.

Single-cell suspensions were processed on the 10X Genomics Chromium iX platform using the Chromium Single Cell 3′ v4 Reagent Kit, following the manufacturer’s instructions for GEM generation, cDNA amplification and library preparation. Approximately 30,000 cells were loaded per run. cDNA yield and library concentration were assessed using Qubit fluorometric quantification, and fragment size distributions were evaluated using an Agilent TapeStation to confirm appropriate cDNA and library profiles prior to sequencing. Sequencing was performed on an Illumina NovaSeq 2000 platform (v1.7.0.45751) with a NovaSeq X Series P4 100-cycle reagent kit. Libraries were sequenced to a target depth of ~20,000 reads per cell, and sequencing outputs were processed using Cell Ranger (v10.0.0) to generate filtered feature-barcode matrices using the GRCm39-2024-A (10X Genomics) reference genome.

Cell Ranger output files (filtered_feature_bc_matrix.h5) from each sample were imported into R (v4.5.2) and assembled into a combined Seurat object (Seurat v5.4.0). Quality control filtering retained cells with 200 – 9,000 detected features and <20% mitochondrial RNA content. Doublets were identified and excluded using scDblFinder (v1.24.0). Data were normalized and variance-stabilized using Seurat’s SCTransform function. Principal component analysis was performed, and the number of principal components explaining 95% of cumulative variance was selected for downstream analysis. Batch effects were corrected using Harmony (v1.2.4). Cell clustering was performed in Seurat using the shared nearest neighbour graph approach with a resolution parameter of 0.6, and cell types were annotated based on marker genes identified by Seurat’s ‘FindAllMarkers’ function (Supplementary Table 3). Immune cells (*Ptprc* >1) were re-analysed separately following the same normalization, dimensionality reduction and clustering workflow (shared nearest neighbour resolution of 0.4). Cell cycle phases were scored using Seurat’s cell cycle functions. Cell–cell communication analysis was performed using CellChat (v2.2.0) on log-normalized RNA assay data, and expression heat maps (*z*-score scaled) were generated using pheatmap (v1.0.13). All additional visualizations were produced with ggplot2 (v4.0.2).

### Induction of neutropenia, anti-IL-17A treatment and anakinra treatment

Neutropenia was induced through the intraperitoneal injection of murinised anti-Ly6G antibody (Absolute Antibodies, Absolute Biotech). On day −1 a loading dose of 300 µg per mouse was administered. Maintenance doses of 150 µg per mouse were administered on day 1 and day 3. Isotype control was administered at the same doses and timepoints. Neutropenia was confirmed in tongue tissue using flow cytometry. Neutropenia was also confirmed in blood extracted by cardiac puncture. In brief, extracted blood was collected into PBS containing 4 mM EDTA and red blood cells lysed using RBC lysis buffer (Biolegend). Cells were washed with PBS, blocked with TruStain FcX (anti-mouse CD16/32) for 15 min at 4 °C and stained with the required antibodies and/or isotypes (Supplementary Table 2) for 30 min at 4 °C. Cells were washed three times with pre-cooled FACS buffer before being run on a BD FACSCanto II (BD Biosciences). Data were collected using BD FACS DIVA (v8.0.1). Results were analysed using FlowJo software (v10.10.0) (BD Biosciences).

IL-17A was blocked through intraperitoneal administration of InVivoMAb anti-IL-17A antibody (Bio X Cell). A dose of 200 µg per mouse was administered on days −1, 1 and 3. Isotype control was administered at the same doses and timepoints. IL-1R was blocked through intraperitoneal administration of anakinra (KINERET, Sobi). A dose of 200 µg per mouse was administered on days −1, 1 and 3.

### Organ collection

Whole brain, liver, kidney, spleen, stomach and caecum were collected and weighed. A 2-cm section of colon was collected and weighed. All tissue was subsequently homogenized using a Gentle MACS (Miltenyi Biotec) and serial dilutions were plated on YPD agar containing 50 μg ml^−1^ chloramphenicol. The plates were cultured at 37 °C for 24 h and c.f.u. was calculated per gram organ.

### Statistical analysis

Data are presented as mean ± s.e.m. or median with interquartile range and are representative or cumulative data from the indicated number of independent experiments. Data were tested for normality and if data followed a Gaussian distribution a one-way ANOVA followed by Bonferroni’s post-test or two-way ANOVA followed by Bonferroni’s post-test was used when multiple groups were analysed. A Kruskal–Wallis test with Dunn’s post-test was used when non-parametric data were analysed. A Gaussian distribution was assumed for experiments with small sample numbers. Data containing zero values was transformed by *Y* = sqrt(*Y* + 0.5).

### Reporting summary

Further information on research design is available in the Nature Portfolio Reporting Summary linked to this article.

## Supplementary information


Reporting Summary
Peer Review File
Supplementary Tables 1, 2 and 3RT-qPCR primers, flow cytometry and blocking antibodies, scRNA-seq cell clustering.


## Source data


Source Data Fig. 1Statistical source data.
Source Data Fig. 2Statistical source data.
Source Data Fig. 3Statistical source data.
Source Data Fig. 5Statistical source data.
Source Data Extended Data Fig. 1Statistical source data.
Source Data Extended Data Fig. 2Statistical source data.
Source Data Extended Data Fig. 3Statistical source data.
Source Data Extended Data Fig. 6Statistical source data.
Source Data Extended Data Fig. 7Statistical source data.


## Data Availability

The RNA-sequencing data have been deposited in ENA at EMBL–EBI under accession number PRJEB61179. The scRNA-seq data have been deposited in ArrayExpress at EMBL–EBI under accession number E-MTAB-16924. Source data are provided with this paper.
