# Supplementary material for: IL-1 family signalling drives mucosal defence against systemic Candida albicans infection
Source: Nat Microbiol. 2026 Aug 5;11(9):2562–76. doi: 10.1038/s41564-026-02431-2 (PMC13524787; doi:10.1038/s41564-026-02431-2)
Supplement: Supplementary file 1 [file 41564_2026_2431_MOESM1_ESM.pdf]

nature portfolio

Corresponding author(s): James S Griffiths

Last updated by author(s): Jun 18, 2026

Reporting Summary

Nature Portfolio wishes to improve the reproducibility of the work that we publish. This form provides structure for consistency and transparency in reporting. For further information on Nature Portfolio policies, see our Editorial Policies and the Editorial Policy Checklist.

Statistics

For all statistical analyses, confirm that the following items are present in the figure legend, table legend, main text, or Methods section.

n/a	Confirmed	
☐	☒	The exact sample size (*n*) for each experimental group/condition, given as a discrete number and unit of measurement
☐	☒	A statement on whether measurements were taken from distinct samples or whether the same sample was measured repeatedly
☐	☒	The statistical test(s) used AND whether they are one- or two-sided<br>*Only common tests should be described solely by name; describe more complex techniques in the Methods section.*
☒	☐	A description of all covariates tested
☐	☒	A description of any assumptions or corrections, such as tests of normality and adjustment for multiple comparisons
☐	☒	A full description of the statistical parameters including central tendency (e.g. means) or other basic estimates (e.g. regression coefficient) AND variation (e.g. standard deviation) or associated estimates of uncertainty (e.g. confidence intervals)
☐	☒	For null hypothesis testing, the test statistic (e.g. *F*, *t*, *r*) with confidence intervals, effect sizes, degrees of freedom and *P* value noted<br>*Give P values as exact values whenever suitable.*
☒	☐	For Bayesian analysis, information on the choice of priors and Markov chain Monte Carlo settings
☒	☐	For hierarchical and complex designs, identification of the appropriate level for tests and full reporting of outcomes
☒	☐	Estimates of effect sizes (e.g. Cohen's *d*, Pearson's *r*), indicating how they were calculated

Our web collection on statistics for biologists contains articles on many of the points above.

Software and code

Policy information about availability of computer code

Data collection	Illumina NovaSeq 2000 v1.7.0.45751 (scRNA-sequencing), BD FACS DIVA v8.0.1 (flow cytometry), Applied Biosystems Quantstudio Real-Time PCR software (v1.7.2).
Data analysis	FlowJo v10.10.0 (flow cytometry), GraphPad Prism v11.0.2 (92) (CFU, weight loss, qPCR, cell numbers, survival and statistical analysis), Kallisto v0.46.1 and R v4.5.2 (RNAseq and scRNA-seq)), 10X Genomics Cell Ranger v10.10.0 (scRNA-seq)

For manuscripts utilizing custom algorithms or software that are central to the research but not yet described in published literature, software must be made available to editors and reviewers. We strongly encourage code deposition in a community repository (e.g. GitHub). See the Nature Portfolio guidelines for submitting code & software for further information.

Data

Policy information about availability of data

All manuscripts must include a data availability statement. This statement should provide the following information, where applicable:

- Accession codes, unique identifiers, or web links for publicly available datasets
- A description of any restrictions on data availability
- For clinical datasets or third party data, please ensure that the statement adheres to our policy

The RNA sequencing data have been deposited in ENA at EMBL-EBI under accession number PRJEB61179. The single-cell RNA sequencing data have been deposited in ArrayExpress at EMBL-EBI under accession number E-MTAB-16924. Source data are provided.

Research involving human participants, their data, or biological material

Policy information about studies with human participants or human data. See also policy information about sex, gender (identity/presentation), and sexual orientation and race, ethnicity and racism.

Reporting on sex and gender	*Use the terms sex (biological attribute) and gender (shaped by social and cultural circumstances) carefully in order to avoid confusing both terms. Indicate if findings apply to only one sex or gender; describe whether sex and gender were considered in study design; whether sex and/or gender was determined based on self-reporting or assigned and methods used. Provide in the source data disaggregated sex and gender data, where this information has been collected, and if consent has been obtained for sharing of individual-level data; provide overall numbers in this Reporting Summary. Please state if this information has not been collected. Report sex- and gender-based analyses where performed, justify reasons for lack of sex- and gender-based analysis.*
Reporting on race, ethnicity, or other socially relevant groupings	*Please specify the socially constructed or socially relevant categorization variable(s) used in your manuscript and explain why they were used. Please note that such variables should not be used as proxies for other socially constructed/relevant variables (for example, race or ethnicity should not be used as a proxy for socioeconomic status). Provide clear definitions of the relevant terms used, how they were provided (by the participants/respondents, the researchers, or third parties), and the method(s) used to classify people into the different categories (e.g. self-report, census or administrative data, social media data, etc.) Please provide details about how you controlled for confounding variables in your analyses.*
Population characteristics	*Describe the covariate-relevant population characteristics of the human research participants (e.g. age, genotypic information, past and current diagnosis and treatment categories). If you filled out the behavioural & social sciences study design questions and have nothing to add here, write "See above."*
Recruitment	*Describe how participants were recruited. Outline any potential self-selection bias or other biases that may be present and how these are likely to impact results.*
Ethics oversight	*Identify the organization(s) that approved the study protocol.*

Note that full information on the approval of the study protocol must also be provided in the manuscript.

Field-specific reporting

Please select the one below that is the best fit for your research. If you are not sure, read the appropriate sections before making your selection.

☒ Life sciences ☐ Behavioural & social sciences ☐ Ecological, evolutionary & environmental sciences

For a reference copy of the document with all sections, see nature.com/documents/nr-reporting-summary-flat.pdf

Life sciences study design

All studies must disclose on these points even when the disclosure is negative.

Sample size	Sample sizes were determined using G*Power analysis where preliminary data were available. Where no preliminary data existed, pilot studies (typically n = 3 per group) were performed to estimate effect sizes. Power calculations assuming 80% power and a significance level of 0.05 (95% confidence) indicated that group sizes of 3 - 12 age- and sex-matched mice were typically sufficient to detect biologically relevant differences including ~1-log changes in fungal burden and ~2-fold changes in gene/protein expression or immunological parameters.
Data exclusions	No data was excluded.
Replication	All experiments were performed in biological duplicate or triplicate depending on the experiment. All attempts at replication were successful.
Randomization	For in vivo experiments investigating genotype-dependent effects without additional intervention, age- and sex-matched control and IL-1RAcP-/- mice were randomly allocated to experimental groups according to experimental duration and/or naïve controls. For in vivo experiments involving additional treatments, mice were randomly allocated to experimental groups using the NC3R's Experimental Design Assistant with genotype and sex incorporated as blocking factors and treatment as the experimental factor.
Blinding	Investigators were blinded to genotype and/or treatment group during in vivo clinical scoring and endpoint assessment. Blinding was not possible for all experiments because treatment groups required distinct interventions and were identifiable during dosing or sample processing. Quantitative readouts, including CFU determination, flow cytometry, qPCR and scRNA-seq analyses, were not performed under blinded conditions.

Reporting for specific materials, systems and methods

We require information from authors about some types of materials, experimental systems and methods used in many studies. Here, indicate whether each material, system or method listed is relevant to your study. If you are not sure if a list item applies to your research, read the appropriate section before selecting a response.

Materials & experimental systems

n/a	Involved in the study
☐	☒ Antibodies
☐	☒ Eukaryotic cell lines
☒	☐ Palaeontology and archaeology
☐	☒ Animals and other organisms
☒	☐ Clinical data
☒	☐ Dual use research of concern
☒	☐ Plants

Methods

n/a	Involved in the study
☒	☐ ChIP-seq
☐	☒ Flow cytometry
☒	☐ MRI-based neuroimaging

Antibodies

Antibodies used	Flow Cytomtery: Anti-mouse CD45 30-F11 (ISO Rat IgG2b κ) Biolegend Catalogue number:103132, Anti-mouse CD4 RM4-5 (ISO Rat IgG2a κ) Biolegend Catalogue number:100531, Anti-mouse TCRβ H57-597 (ISO Armenian Hamster IgG) Biolegend Catalogue number:109212, Anti-mouse TCRγδ GL3 (ISO Armenian Hamster IgG) Biolegend Catalogue number:118108, Anti-mouse CD11b M1/70 (ISO Rat IgG2b κ) Biolegend Catalogue number:101224, Anti-mouse Ly6G 1A8 (ISO Rat IgG2a κ) Biolegend Catalogue number:127608, Anti-mouse Ly6C HK1.4 (ISO Rat IgG2c κ) Biolegend Catalogue number:128016, Anti-mouse GR-1 RB6-8C5 (ISO Rat IgG2b κ) Biolegend Catalogue number:108424, Anti-mouse CD101 Moushi101 (Rat IgG2a κ) Invitrogen Catalogue number:25-1011-82.<br><br>Blocking/Depletion: Vivopure X Anti-Ly6G 1A8 (ISO Mouse IgG2a) Absolute Antibody Catalogue number:AB00295-2.0-VXM, InVivoMAb anti-mouse/rat IL-17A 17F3 (ISO Mouse IgG1 κ) Bio X Cell Catalogue number:BE0173-5MG
Validation	Antibodies were used according to the manufacturers' recommendations and assessed using appropriate isotype controls and established gating strategies (Extended Data Figure 2c, Extended Data Figure 3c-d, f-g, Extended Data Figure 6a-b). Antibody specificity and application suitability were supported by manufacturers' (BioLegend, Invitrogen and Bio X Cell) validation data.

Eukaryotic cell lines

Policy information about cell lines and Sex and Gender in Research

Cell line source(s)	Commercially available: TR146 Human Oral Epithelial Cell line. Derived from buccal carcinoma of 67-year old female patient. acquired from Cancer Tools (catalogue number: 151425) and the European Collection of Authenticated Cell Cultures (catalogue number: 10032305).
Authentication	Cell line was not authenticated.
Mycoplasma contamination	Mycoplasma was routinely (~ every 3 months) tested for and cells were negative.
Commonly misidentified lines (See ICLAC register)	*Name any commonly misidentified cell lines used in the study and provide a rationale for their use.*

Animals and other research organisms

Policy information about studies involving animals; ARRIVE guidelines recommended for reporting animal research, and Sex and Gender in Research

Laboratory animals	B6;129S1-Il1raptm1Roml/J were purchased from Jax (RRID:IMSR_JAX:003284) and backcrossed for 10 generations to C57BL/6J (RRID:IMSR_JAX:000664). Throughout the study IL1RAcP-/- represent the backcrossed B6;129S1-Il1raptm1Roml/J, and control mice represent C57BL/6J. Male and female 8-12 week old mice were used in experimentation.
Wild animals	Wild animals were not used.
Reporting on sex	Experiments were performed in male and female mice.
Field-collected samples	Field-collected samples were not used in this study.
Ethics oversight	Mice were maintained and used according to King's College London institutional and UK Home Office guidelines. This study was performed in strict accordance with the Project License (PP6711863). The animal care and use protocol adhered to the Animals (Scientific Procedures) Act 1986.

Note that full information on the approval of the study protocol must also be provided in the manuscript.

Plants

Seed stocks

Report on the source of all seed stocks or other plant material used. If applicable, state the seed stock centre and catalogue number. If plant specimens were collected from the field, describe the collection location, date and sampling procedures.

Novel plant genotypes

Describe the methods by which all novel plant genotypes were produced. This includes those generated by transgenic approaches, gene editing, chemical/radiation-based mutagenesis and hybridization. For transgenic lines, describe the transformation method, the number of independent lines analyzed and the generation upon which experiments were performed. For gene-edited lines, describe the editor used, the endogenous sequence targeted for editing, the targeting guide RNA sequence (if applicable) and how the editor was applied.

Authentication

Describe any authentication procedures for each seed stock used or novel genotype generated. Describe any experiments used to assess the effect of a mutation and, where applicable, how potential secondary effects (e.g. second site T-DNA insertions, mosiacism, off-target gene editing) were examined.

Flow Cytometry

Plots

Confirm that:

☒ The axis labels state the marker and fluorochrome used (e.g. CD4-FITC).

☒ The axis scales are clearly visible. Include numbers along axes only for bottom left plot of group (a 'group' is an analysis of identical markers).

☐ All plots are contour plots with outliers or pseudocolor plots.

☒ A numerical value for number of cells or percentage (with statistics) is provided.

Methodology

Sample preparation

Half tongue was collected on ice and homogenised using a Gentle MACS (Miltenyi Biotec) with a tumour dissociation kit (Miltenyi Biotec). Single cell homogenate was passed through a 70 µm filter and washed with pre-cooled RPMI 1640 without phenol red (Gibco, ThermoFisher) before being counted and resuspended in pre-cooled PBS. Cells were live/dead stained with Zombie Green Fixable Viability Kit (Biolegend) for 30 min at 4oC and then washed in pre-cooled FACS Buffer (PBS 3% FBS 2 mM EDTA). Cells were blocked with TruStain FcX (anti-mouse CD16/32) (Biolegend) for 15 min at 4oC before being stained with the required antibodies and/or isotypes for 30 min at 4oC. Cells were washed three times with pre-cooled FACS buffer before being run on a BD FACSCanto II (BD Biosciences). Results were analysed using FlowJo software (BD Biosciences).

Extracted blood was collected into PBS containing 4 mM EDTA and red blood cells lysed using RBC lysis buffer (Biolegend). Cells were washed with PBS, blocked with TruStain FcX (anti-mouse CD16/32) for 15 min at 4oC and stained with the required antibodies and/or isotypes for 30 min at 4oC. Cells were washed three times with pre-cooled FACS buffer before being run on a BD FACSCanto II (BD Biosciences). Results were analysed using FlowJo software (BD Biosciences).

Instrument

BD FACS Canto II

Software

FlowJo V10 was used to analyse results. GraphPad Prism was used to make graphs and complete statistical anaylsis.

Cell population abundance

The percentages shown are based on % live cells. neutrophils were identfied by their expression of CD45, Ly6G and Cd11b. ab T cells were identified by their expression of CD45, CD4, ab TCR. gd T cells were identified by their expression of CD45, CD4, gd TCR.

Gating strategy

Gating strategy for flow cytometry analysis included an FSC SSC initial gate, a live selection gate, a single cell gate, a CD45+ gate and then further cell-type-dependent downstream analysis.

☒ Tick this box to confirm that a figure exemplifying the gating strategy is provided in the Supplementary Information.
