# Supplementary material for: IL-1 family signalling drives mucosal defence against systemic Candida albicans infection
Source: Nat Microbiol. 2026 Aug 5;11(9):2562–76. doi: 10.1038/s41564-026-02431-2 (PMC13524787; doi:10.1038/s41564-026-02431-2)
Supplement: Supplementary file 2 [file 41564_2026_2431_MOESM2_ESM.pdf]

nature portfolio

Peer Review File

IL-1 family signalling drives mucosal defense against systemic Candida albicans infection

Corresponding Author: Dr James Griffiths

Version 0:

Reviewer comments:

Reviewer #1

(Remarks to the Author)
This is a well-executed study that investigates the importance of IL-1R signaling in the early stages of oropharyngeal candidiasis and in controlling systemic fungal dissemination in IL-1R-deficient setting. The study underscore the role of IL-1 family on protection against fungal dissemination during OPC and on how neutrophils prevent life-threatening systemic infections in this setting.
A key mechanistic question is: which cell types require IL-1 signaling in the absence of neutrophils and ultimately prevent dissemination? The data show that IL-1 signaling is crucial during the early phase of infection. Testing experimentally or at least analyzing cell types where IL-1R is essential to prevent systemic candidiasis during neutropenia in OPC would substantiate this model. Such data could further help explaining why IL-1R blockade with anakinra did not result in dissemination.
The finding that the ece1Δ/Δ strain, despite colonizing mucosal tissues comparably to WT, fails to disseminate systemically is intriguing. The data strongly suggest that candidalysin is essential for fungal dissemination. Is candidalysin essential once the systemic dissemination has occurred or is it necessary earlier for the penetration of the oral mucosa?
It will strengthen the manuscript if the authors contextualize these findings alongside previous work. For instance, PMID: 29101209 demonstrated OEC as a source of IL-1 in the OPC model triggered by candidalysin. PMID: 35296857 demonstrated a protective role of anakinra in the context of intestinal inflammation aggravated by C. albicans and candidalysin. Interestingly, in the current study, anakinra doesn't confer protection in OPC, which highlights the complexity and context-specific nature of IL-1 signaling. Drawing these parallels could provide important perspective on how IL1R, or its blockade, can have divergent effects depending on mucosal tissue site and model used.

Overall, this is an insightful study that adds important mechanistic detail to the role of IL-1 signaling and candidalysin in OPC and systemic candidiasis. Addressing the points above would further strengthen its impact.

Reviewer #2

(Remarks to the Author)
This paper analyses the phenotype of IL-1RAcP-/- KO mice when challenged with the fungus Candida in a mucosal infection model. They find that the KO mice have worse control of the infection within 24 h of infection, but this resolves similarly to WT mice alongside an exaggerated immune response at 48h post infection. The authors then deplete neutrophils in their animals to mimic the main risk factor for this disease, neutropenia. Strikingly, they find the KO mice begin to succumb to the mucosal infection, with extensive dissemination to the liver and other organs which is unusual in this model. Their data therefore shows that neutrophils and IL-1 signalling co-operate for early antifungal immunity in the oral mucosa and contain the fungus.

Overall, I thought this was nice paper with new insights and was very well-presented and written. The data is high quality and reproducible. However, I felt the paper stopped short and I was hoping to learn more about the mechanisms underlying their observation. For example, what cells in the mucosa are producing IL-1 and what cells are responding to it to make the neutrophil chemoattractants? How are neutrophils preventing dissemination to the liver – just by killing fungi in the mucosa? Why don't we see effects in the WT neutropenic mice in that case? Joining the dots up a bit would increase the impact of the work.

As such, I feel this paper represents an important new insight for the field and generates several outstanding remaining questions. My specific points/questions below.

1. IL-17/22/23 are all elevated in the KO mice at day 2 post-infection compared to WT. Is this linked to higher CFU in the KO mice? Is there a positive correlation between the 2 variables? Put another way, would you expect to see increased cytokines with increased CFU in WT mice, suggesting that induction of cytokines is running normally rather than an abnormal supra-induction in the absence of IL-1?
2. Fig 2c; what are the FACS plots gated on? What is the population in the middle – the legend would suggest these are T-cells

without a TCR? How can that be?
3. The authors conclude that once T cell infiltration is established, there is no dependence on IL-1 family. However, have the authors ruled out an effect on local proliferation to determine whether there are additional influences here, rather than just on infiltration? For example, could low numbers infiltrate and then expand in the tissue, and this is just delayed in the KO mice?
4. Frequency of neutrophils is shown, but it would be better to show total numbers, particularly given that there are differences in lymphocytes (and possibly proliferation?) that may skew proportions of cells as determined by flow cytometry. Quantification of their histology images may also be a help here.
5. Does CARD9 play a role in the IL-1-PMN pathway? Analysis of CARD9 involvement could help explain some of the susceptibility to OPC in CARD9 deficiency and would nicely link up with previous work on IL-1-driven CARD9-dependent neutrophil recruitment in systemic infection.
6. Lines 327-329; slightly unfair comment given that IV infection models are good models of catheter related disease in which kidney (rather than liver) is primarily infected.
7. Data with Anakinra in Fig 4 is somewhat at odds with the data using KO mice (and this perhaps requires more explanation/discussion). Wouldn't we expect the drug to block the IL-1 receptor and therefore most of IL-1 signalling, and would therefore mimic the KO mice phenotype? Which aspect of IL-1 signalling isn't being blocked here and can that be leveraged to understand the mechanism in more detail?

Reviewer #3

(Remarks to the Author)
In this study by Griffiths, et al the authors investigated the impact of IL-1 family cytokines to defense against mucosal Candida infection in the oral cavity. Oral Candida infections (thrush) affect millions of people worldwide. In this study the authors present exciting new data to support the conclusion that cooperation between IL-1 family cytokines and neutrophils is needed to contain Candida infection in the oral cavity and prevent dissemination. They also further identify a key role for the virulence factor candidalysin in triggering expression of IL-1 cytokines as well as being required for dissemination in immune-deficient mice (defective in both IL-1 cytokine sensing and neutrophils). The authors determined that stimulation of a human oral epithelial cell line with Candida resulted in the induction of various IL-1 family cytokines and that this induction was dependent on the virulence factor candidalysin. To investigate the impact of IL-1 family cytokines to control of oral Candida infection in vivo the authors employed IL-1RAcp-deficient mice where this deficiency results in impaired activation of multiple IL-1 family cytokines including IL-1a, IL-1b, IL-33 and IL-36 family members. In mice defective in IL-1 family responses there was a significant, but transient effect (day 2), in control of Candida infection that was resolved by day 3 of infection. At day 2 after oral infection, IL-1 deficiency affected multiple host response pathways that are known to be important for Candida containment, these included: reduced expression of antimicrobial peptides, reduced IL-17 expression and reduced recruitment of neutrophils and T cells. By day 3 after infection, control animals and IL-1RAcp-/- mice had cleared the infection (Fig 1c) which correlated with a robust infiltration of neutrophils (Fig 3). The authors tested the impact of the exuberant infiltration of neutrophils seen in IL-1RAcp-/- to control of infection by depleting neutrophils with anti-Ly6G antibodies. Strikingly, neutrophil-depleted, IL-1 deficient mice succumbed to oral infection (Fig 4a) and developed systemic infection (Figs 4b-d). They further show that candidalysin was a key factor required for dissemination where infection of IL-1-defective, neutropenic mice with a candidalysin-deficient Candida strain failed to disseminate or cause mortality. Altogether, the findings are novel, and the data presented support the key conclusions presented. My questions and recommendations for the authors are:

1) Neutrophil depletion is an important approach for the mechanistic data shown in in figure 4. The use of anti-Ly6G antibodies is a well-accepted method to achieve neutrophil depletion in vivo. However, there are technical challenges with the approach including concerns with partial effectiveness, variable impact to neutrophil depletion in an organ-specific manner as well as technical issues with properly detecting neutrophil depletion due to antigen masking by 1A8-bound cells that appear depleted in flow cytometry especially when the staining is done with the same clone used for depletion (PMID: 31226358). Based on the data shown in supplementary figure 4 it appears that the authors confirmed depletion by staining with Ly6G. Did the authors employ alternative methods to ensure proper depletion? Some options include staining with Gr1 or intracellular staining for Ly6G (PMID: 32488020).
2) The authors mentioned in their discussion that they "identified an influx of immature neutrophils, suggesting not only that emergency granulopoiesis can be initiated independently of the IL-1 family, but that the IL-1RAcP-/- model required substantial neutrophil infiltration to combat severe OPC infection." This is an interesting idea. Based on data shown in supplementary figure 3 it appears that the only metric used for identifying immature neutrophils was variable Ly6G levels. Did the authors test other important maturation markers like CXCR2 and CD101?
3) Neutrophil depletion in IL-1RAcP-/- resulted in some intriguing effects including reduced Il-17a expression and even reduced infiltration of T cells. The authors nicely discuss how neutrophils could affect IL-17 expression based on previous work. Do they have any thoughts on how neutropenia affected T cell recruitment?

Reviewer #4

(Remarks to the Author)
This manuscript describes the critical role for the IL-1 signaling axis in protective immune responses during mucosal candidiasis (OPC). The authors discover that IL-1 signaling (namely IL-1RAcP-depedent signaling) occurs as a result of candidalysin induced host damage during early mucosal Candida infection, which triggers IL-1 mediated recruitment of neutrophils, TCRab+, and TCRgd+ cells that help control fungal growth within the mucosa and fungal dissemination to secondary organs. The data itself is interesting, and proper controls are used in most experiments throughout the manuscript, but the study as a whole is not particularly novel (IL-1 signaling importance is candidiasis has been described) and is lacking mechanistic detail to strengthen the conclusions built on predominantly observational data. To be published in Nature Microbiology, this study requires confirmatory mechanistic analyses to concretely tie the IL-1 signaling axis to the observed findings of reduced immune cell

populations to the lack of fungal control. These mechanistic analyses required for publication in Nature Microbiology may not be required for a lower impact journal.

Major Critiques:

Study not particularly novel and lacking mechanistic analysis required to concretely tie observations together: This manuscript although well executed and interesting is lacking a bit of novelty and impact for publication in Nature Microbiology. The importance of the IL-1 axis in candidiasis has been described by this group and others (PMID: 37478856; 29891557).

Secondly, in the present study mechanistic ties between IL-1RAcP, immune cell abundance in the oral mucosa (neutrophils, T cells, etc.), and diminished fungal control and dissemination are strictly observational connections. More concrete mechanistic study will be needed to fill the gaps between these observations and support IL-1 signaling dependence and sufficiency.

More detailed analysis and discussion of IL-1RAcP is needed: Importantly, in the introduction describe why the knockout in this specific gene is needed to study IL-1 signaling defects and not other genes in mucosal candidiasis. This is described in the results (L119-121) but is missing in the introduction and is necessary to set the stage. Also, is it known whether patients with variants in IL-1RAcP gene (or other IL-1 signaling axis components) are at increased susceptibility for disease? If there are, this would greatly strengthen the clinical relevance of your research.

Additionally, it would be important to establish the time and cell-type dependent role of IL-1RAcP during mucosal candidiasis. Which cells are known to express IL-1RAcP in the oral mucosa and are likely to response to this IL-1 signal? How is its expression altered throughout the course of infection? Lineage tracing experiments during wildtype OPC would be invaluable to set the stage for who/what (can and is responding to these signals), when (are these signals acting), and where IL-1RAcP is acting in the context of mucosal candidiasis.

Relevance to Patient Populations: When might IL-1 signaling defects be seen in the course of natural infection in humans? Is diminished IL-1 signaling present in susceptible patient populations? Is it known whether neutropenic patients have reduced IL-1 signaling or conversely are patients on anakinra, canakinumab, and rilonacept at increased risk for uncontrolled mucosal candidiasis? The introduction could benefit from the addition of the clinical relevance of exploring the IL-1 axis in disease.

Minor Critiques:

Cytokine and chemokine levels throughout should be measured at the protein level.

Could you display flow data in both % total live cell and absolute number/g tissue?

L35: please move “risk” after “patient” and before “factor” in this sentence.

L127: The time frame for the early time point is unclear. “Immediately” and “within 1 day” are hard to interpret. Can you describe this is hours (<X hours)?

L159: Please add a reference for the scRNAseq study or in situ study for “given that beta-defensin 3 is epithelial-derived.”

L166-167: Natural TCRab+ and TCRgd+ cells are described in this section, but the data presented doesn’t confirm that these are the cells identified in this study. Please confirm this phenotype or remove description of “natural” TCRab+ and TCRgd+ as this is not supported. Additionally, are these cells the source of the IL-17 production and dependent on IL-1 signaling? This would help fill one mechanism gap.

L170: “indicating that cellular recruitment” This statement should be confirmed by checking that proliferation of these cells isn’t the reason for the increased abundance of cells.

L175: “their absence” I’m not sure if this is accurate. Consider revising this statement, because the data indicates that this is lower at day 1, but higher at day 2.

L183 and Figure 3a: The histology images presented here do not seem as severe as the pathology seen in Figure 1. I understand one is PAS and the other is H&E, but the tissue layers are different. Is there a spectrum of disease and disease kinetics in the IL-1RAcP-/- mouse?

L186: “However, by day 2. . .” What signals might be compensating for the IL-1 at this later time frame? IL-1 seems to be very time-period dependent throughout this manuscript. Also, in the absence of candidalysin (ece-/-) is this recruitment missing and chemokines not expressed?

L219: A clear rationale is needed for this set of experiments. If TCRab+ cell recruitment/proliferation is delayed in IL-1RAcP-/- mice and the hypothesis is that neutrophils are providing the signal to bring them in/induce proliferation, then to support this connection mechanistically you would want to design an experiment that results in restoration of this recruitment in IL-1RAcP deficient mice. Based on the presented data, it is unclear what cell types and what signals are minimally required to bring about this correction in OPC disease.

Figure 4b-d: What was seen for both mouse genotypes in the isotype control experiments for these parameters? Is this the result of Ly6G depletion and IL-1 signaling deficiency or can it be seen in IL-1 signaling deficiency alone?

Figure 4i: What would occur in a mouse treated with anakinara and Ly6G depletion? Similar to Figure 4b-d results? This experiment would help support findings above.

Decision Letter:

16th May 2025

*Please ensure you delete the link to your author homepage in this e-mail if you wish to forward it to your co-authors.

Dear James and Julian,

Thank you for your patience while your manuscript "The IL-1 Family Controls Acute Mucosal Fungal Infection and Mucosal-Systemic Dissemination." was under peer-review at Nature Microbiology. It has now been seen by 4 referees, whose expertise and comments you will find at the end of this email. Although they find your work of some potential interest, they have raised a number of concerns that will need to be addressed before we can consider publication of the work in Nature Microbiology.

In particular, referee #1 suggests testing experimentally or at least analyzing cell types where IL-1R is essential to prevent systemic candidiasis during neutropenia in OPC. The referee also says the findings need to be better contextualized alongside previous work. Referee #2 asks for some more mechanistic insights, and asks what cells in the mucosa are producing IL-1 and what cells are responding to it to make the neutrophil chemo-attractants. Further, the referee asks how neutrophils are preventing dissemination to the liver, and why we don't see effects in the WT neutropenic mice. Referee #3 has some questions for technical clarification. Referee #4 feels the study is not particularly novel and is lacking mechanistic detail to strengthen the conclusions. The referee says that mechanistic analyses should be performed to tie the IL-1 signaling axis to the observed findings of reduced immune cell populations to the lack of fungal control. The referee also feels that more detailed analysis and discussion of IL-1RAcP is needed, and that it would be important to establish the time and cell-type dependent role of IL-1RAcP during mucosal candidiasis. Please note that we editorially overrule referee #4's concern over novelty, but that we will need the remaining referee concerns to be addressed.

Should further experimental data allow you to address these criticisms, we would be happy to look at a revised manuscript.

We are committed to providing a fair and constructive peer-review process. Please do not hesitate to contact us if there are specific requests from the reviewers that you believe are technically impossible or unlikely to yield a meaningful outcome.

We strongly support public availability of data. Please place the data used in your paper into a public data repository, if one exists, or alternatively, present the data as Source Data or Supplementary Information. If data can only be shared on request, please explain why in your Data Availability Statement, and also in the correspondence with your editor. For some data types, deposition in a public repository is mandatory - more information on our data deposition policies and available repositories can be found at https://www.nature.com/nature-research/editorial-policies/reporting-standards#availability-of-data.

Please include a data availability statement as a separate section after Methods but before references, under the heading "Data Availability". This section should inform readers about the availability of the data used to support the conclusions of your study. This information includes accession codes to public repositories (data banks for protein, DNA or RNA sequences, microarray, proteomics data etc…), references to source data published alongside the paper, unique identifiers such as URLs to data repository entries, or data set DOIs, and any other statement about data availability. At a minimum, you should include the following statement: "The data that support the findings of this study are available from the corresponding author upon request", mentioning any restrictions on availability. If DOIs are provided, we also strongly encourage including these in the Reference list (authors, title, publisher (repository name), identifier, year). For more guidance on how to write this section please see: http://www.nature.com/authors/policies/data/data-availability-statements-data-citations.pdf

When revising your manuscript:

* Include a "Response to referees" document detailing, point-by-point, how you addressed each referee comment. If no action was taken to address a point, you must provide a compelling argument. This response will be sent back to the referees along with the revised manuscript.

* If you have not done so already we suggest that you begin to revise your manuscript so that it conforms to our Article format instructions at http://www.nature.com/nmicrobiol/info/final-submission. Refer also to any guidelines provided in this letter.

* Include a revised version of any required reporting checklist. It will be available to referees (and, potentially, statisticians) to aid in their evaluation if the manuscript goes back for peer review. A revised checklist is essential for re-review of the paper.

When submitting the revised version of your manuscript, please pay close attention to our href="https://www.nature.com/nature-portfolio/editorial-policies/image-integrity">Digital Image Integrity Guidelines. and to the following points below:

-- that unprocessed scans are clearly labelled and match the gels and western blots presented in figures.
-- that control panels for gels and western blots are appropriately described as loading on sample processing controls
-- all images in the paper are checked for duplication of panels and for splicing of gel lanes.

EXTENDED DATA FIGURES

When re-submitting your manuscript, please ensure that any supplementary figures and tables that are crucial to the manuscript's conclusions are converted into Extended Data figures and tables to increase visibility of these data. Extended Data figures and tables are online-only (present in the online PDF and full-text HTML versions of the paper), peer-reviewed display items that provide essential background to the article but are not included in the main article due to space constraints. A maximum of ten Extended Data display items (figures and tables) is permitted.

Finally, please ensure that you retain unprocessed data and metadata files after publication, ideally archiving data in perpetuity, as these may be requested during the peer review and production process or after publication if any issues arise.

Please use the link below to submit a revised paper:

Link Redacted

Note: This url links to your confidential homepage and associated information about manuscripts you may have submitted or be reviewing for us. If you wish to forward this e-mail to co-authors, please delete this link to your homepage first.

Nature Microbiology is committed to improving transparency in authorship. As part of our efforts in this direction, we are now requesting that all authors identified as 'corresponding author' on published papers create and link their Open Researcher and Contributor Identifier (ORCID) with their account on the Manuscript Tracking System (MTS), prior to acceptance. This applies to primary research papers only. ORCID helps the scientific community achieve unambiguous attribution of all scholarly contributions. You can create and link your ORCID from the home page of the MTS by clicking on 'Modify my Springer Nature account'. For more information please visit please visit www.springernature.com/orcid.

If you wish to submit a suitably revised manuscript we would hope to receive it within 6 months. If you cannot send it within this time, please let us know.

In the meantime we hope that you find our referees' comments helpful.

Yours sincerely,

****
Reviewer Expertise:

Referee #1: Fungal immunology, mucosal immunology
Referee #2: Fungal immunology, Candida albicans
Referee #3: Host immune responses
Referee #4: Host immune responses

Reviewer Comments:

Reviewer #1 (Remarks to the Author):

This is a well-executed study that investigates the importance of IL-1R signaling in the early stages of oropharyngeal candidiasis and in controlling systemic fungal dissemination in IL-1R-deficient setting. The study underscore the role of IL-1 family on protection against fungal dissemination during OPC and on how neutrophils prevent life-threatening systemic infections in this setting.
A key mechanistic question is: which cell types require IL-1 signaling in the absence of neutrophils and ultimately prevent dissemination? The data show that IL-1 signaling is crucial during the early phase of infection. Testing experimentally or at least analyzing cell types where IL-1R is essential to prevent systemic candidiasis during neutropenia in OPC would substantiate this model. Such data could further help explaining why IL-1R blockade with anakinra did not result in dissemination.
The finding that the ece1Δ/Δ strain, despite colonizing mucosal tissues comparably to WT, fails to disseminate systemically is intriguing. The data strongly suggest that candidalysin is essential for fungal dissemination. Is candidalysin essential once the systemic dissemination has occurred or is it necessary earlier for the penetration of the oral mucosa?
It will strengthen the manuscript if the authors contextualize these findings alongside previous work. For instance, PMID: 29101209 demonstrated OEC as a source of IL-1 in the OPC model triggered by candidalysin. PMID: 35296857 demonstrated a protective role of anakinra in the context of intestinal inflammation aggravated by C. albicans and candidalysin. Interestingly, in the current study, anakinra doesn't confer protection in OPC, which highlights the complexity and context-specific nature of IL-1 signaling. Drawing these parallels could provide important perspective on how IL1R, or its blockade, can have divergent effects depending on mucosal tissue site and model used.

Overall, this is an insightful study that adds important mechanistic detail to the role of IL-1 signaling and candidalysin in OPC and systemic candidiasis. Addressing the points above would further strengthen its impact.

Reviewer #2 (Remarks to the Author):

This paper analyses the phenotype of IL-1RAcP-/- KO mice when challenged with the fungus Candida in a mucosal infection model. They find that the KO mice have worse control of the infection within 24 h of infection, but this resolves similarly to WT mice alongside an exaggerated immune response at 48h post infection. The authors then deplete neutrophils in their animals to mimic the main risk factor for this disease, neutropenia. Strikingly, they find the KO mice begin to succumb to the mucosal infection, with extensive dissemination to the liver and other organs which is unusual in this model. Their data therefore shows that neutrophils and IL-1 signalling co-operate for early antifungal immunity in the oral mucosa and contain the fungus.

Overall, I thought this was nice paper with new insights and was very well-presented and written. The data is high quality and reproducible. However, I felt the paper stopped short and I was hoping to learn more about the mechanisms underlying their observation. For example, what cells in the mucosa are producing IL-1 and what cells are responding to it to make the neutrophil chemoattractants? How are neutrophils preventing dissemination to the liver – just by killing fungi in the mucosa? Why don't we see effects in the WT neutropenic mice in that case? Joining the dots up a bit would increase the impact of the work.

As such, I feel this paper represents an important new insight for the field and generates several outstanding remaining questions. My specific points/questions below.

1. IL-17/22/23 are all elevated in the KO mice at day 2 post-infection compared to WT. Is this linked to higher CFU in the KO mice? Is there a positive correlation between the 2 variables? Put another way, would you expect to see increased cytokines with increased CFU in WT mice, suggesting that induction of cytokines is running normally rather than an abnormal supra-induction in the absence of IL-1?
2. Fig 2c; what are the FACS plots gated on? What is the population in the middle – the legend would suggest these are T-cells without a TCR? How can that be?
3. The authors conclude that once T cell infiltration is established, there is no dependence on IL-1 family. However, have the authors ruled out an effect on local proliferation to determine whether there are additional influences here, rather than just on infiltration? For example, could low numbers infiltrate and then expand in the tissue, and this is just delayed in the KO mice?
4. Frequency of neutrophils is shown, but it would be better to show total numbers, particularly given that there are differences in lymphocytes (and possibly proliferation?) that may skew proportions of cells as determined by flow cytometry. Quantification of their histology images may also be a help here.
5. Does CARD9 play a role in the IL-1-PMN pathway? Analysis of CARD9 involvement could help explain some of the susceptibility to OPC in CARD9 deficiency and would nicely link up with previous work on IL-1-driven CARD9-dependent neutrophil recruitment in systemic infection.
6. Lines 327-329; slightly unfair comment given that IV infection models are good models of catheter related disease in which kidney (rather than liver) is primarily infected.
7. Data with Anakinra in Fig 4 is somewhat at odds with the data using KO mice (and this perhaps requires more explanation/discussion). Wouldn't we expect the drug to block the IL-1 receptor and therefore most of IL-1 signalling, and would therefore mimic the KO mice phenotype? Which aspect of IL-1 signalling isn't being blocked here and can that be leveraged to understand the mechanism in more detail?

Reviewer #3 (Remarks to the Author):

In this study by Griffiths, et al the authors investigated the impact of IL-1 family cytokines to defense against mucosal Candida infection in the oral cavity. Oral Candida infections (thrush) affect millions of people worldwide. In this study the authors present exciting new data to support the conclusion that cooperation between IL-1 family cytokines and neutrophils is needed to contain Candida infection in the oral cavity and prevent dissemination. They also further identify a key role for the virulence factor candidalysin in triggering expression of IL-1 cytokines as well as being required for dissemination in immune-deficient mice (defective in both IL-1 cytokine sensing and neutrophils). The authors determined that stimulation of a human oral epithelial cell line with Candida resulted in the induction of various IL-1 family cytokines and that this induction was dependent on the virulence factor candidalysin. To investigate the impact of IL-1 family cytokines to control of oral Candida infection in vivo the authors employed IL-1RAcp-deficient mice where this deficiency results in impaired activation of multiple IL-1 family cytokines including IL-1a, IL-1b, IL-33 and IL-36 family members. In mice defective in IL-1 family responses there was a significant, but transient effect (day 2), in control of Candida infection that was resolved by day 3 of infection. At day 2 after oral infection, IL-1 deficiency affected multiple host response pathways that are known to be important for Candida containment, these included: reduced expression of antimicrobial peptides, reduced IL-17 expression and reduced recruitment of neutrophils and T cells. By day 3 after infection, control animals and IL-1RAcp-/- mice had cleared the infection (Fig 1c) which correlated with a robust infiltration of neutrophils (Fig 3). The authors tested the impact of the exuberant infiltration of neutrophils seen in IL-1RAcp-/- to control of infection by depleting neutrophils with anti-Ly6G antibodies. Strikingly, neutrophil-depleted, IL-1 deficient mice succumbed to oral infection (Fig 4a) and developed systemic infection (Figs 4b-d). They further show that candidalysin was a key factor required for dissemination where infection of IL-1-defective, neutropenic mice with a candidalysin-deficient Candida strain failed to disseminate or cause mortality. Altogether, the findings are novel, and the data presented support the key conclusions presented. My questions and recommendations for the authors are:

1) Neutrophil depletion is an important approach for the mechanistic data shown in in figure 4. The use of anti-Ly6G antibodies is a well-accepted method to achieve neutrophil depletion in vivo. However, there are technical challenges with the approach including concerns with partial effectiveness, variable impact to neutrophil depletion in an organ-specific manner as well as technical issues with properly detecting neutrophil depletion due to antigen masking by 1A8-bound cells that appear depleted in flow cytometry especially when the staining is done with the same clone used for depletion (PMID: 31226358). Based on the data shown in supplementary figure 4 it appears that the authors confirmed depletion by staining with Ly6G. Did the authors employ alternative methods to ensure proper depletion? Some options include staining with Gr1 or intracellular staining for Ly6G (PMID: 32488020).

2) The authors mentioned in their discussion that they “identified an influx of immature neutrophils, suggesting not only that emergency granulopoiesis can be initiated independently of the IL-1 family, but that the IL-1RAcP-/- model required substantial neutrophil infiltration to combat severe OPC infection.” This is an interesting idea. Based on data shown in supplementary figure 3 it appears that the only metric used for identifying immature neutrophils was variable Ly6G levels. Did the authors test other important maturation markers like CXCR2 and CD101?
3) Neutrophil depletion in IL-1RAcP-/- resulted in some intriguing effects including reduced Il-17a expression and even reduced infiltration of T cells. The authors nicely discuss how neutrophils could affect IL-17 expression based on previous work. Do they have any thoughts on how neutropenia affected T cell recruitment?

Reviewer #4 (Remarks to the Author):

This manuscript describes the critical role for the IL-1 signaling axis in protective immune responses during mucosal candidiasis (OPC). The authors discover that IL-1 signaling (namely IL-1RAcP-depedent signaling) occurs as a result of candidalysin induced host damage during early mucosal Candida infection, which triggers IL-1 mediated recruitment of neutrophils, TCRab+, and TCRgd+ cells that help control fungal growth within the mucosa and fungal dissemination to secondary organs. The data itself is interesting, and proper controls are used in most experiments throughout the manuscript, but the study as a whole is not particularly novel (IL-1 signaling importance is candidiasis has been described) and is lacking mechanistic detail to strengthen the conclusions built on predominantly observational data. To be published in Nature Microbiology, this study requires confirmatory mechanistic analyses to concretely tie the IL-1 signaling axis to the observed findings of reduced immune cell populations to the lack of fungal control. These mechanistic analyses required for publication in Nature Microbiology may not be required for a lower impact journal.

Major Critiques:

Study not particularly novel and lacking mechanistic analysis required to concretely tie observations together: This manuscript although well executed and interesting is lacking a bit of novelty and impact for publication in Nature Microbiology. The importance of the IL-1 axis in candidiasis has been described by this group and others (PMID: 37478856; 29891557).

Secondly, in the present study mechanistic ties between IL-1RAcP, immune cell abundance in the oral mucosa (neutrophils, T cells, etc.), and diminished fungal control and dissemination are strictly observational connections. More concrete mechanistic study will be needed to fill the gaps between these observations and support IL-1 signaling dependence and sufficiency.

More detailed analysis and discussion of IL-1RAcP is needed: Importantly, in the introduction describe why the knockout in this specific gene is needed to study IL-1 signaling defects and not other genes in mucosal candidiasis. This is described in the results (L119-121) but is missing in the introduction and is necessary to set the stage. Also, is it known whether patients with variants in IL-1RAcP gene (or other IL-1 signaling axis components) are at increased susceptibility for disease? If there are, this would greatly strengthen the clinical relevance of your research.

Additionally, it would be important to establish the time and cell-type dependent role of IL-1RAcP during mucosal candidiasis. Which cells are known to express IL-1RAcP in the oral mucosa and are likely to response to this IL-1 signal? How is its expression altered throughout the course of infection? Lineage tracing experiments during wildtype OPC would be invaluable to set the stage for who/what (can and is responding to these signals), when (are these signals acting), and where IL-1RAcP is acting in the context of mucosal candidiasis.

Relevance to Patient Populations: When might IL-1 signaling defects be seen in the course of natural infection in humans? Is diminished IL-1 signaling present in susceptible patient populations? Is it known whether neutropenic patients have reduced IL-1 signaling or conversely are patients on anakinra, canakinumab, and rilonacept at increased risk for uncontrolled mucosal candidiasis? The introduction could benefit from the addition of the clinical relevance of exploring the IL-1 axis in disease.

Minor Critiques:

Cytokine and chemokine levels throughout should be measured at the protein level.

Could you display flow data in both % total live cell and absolute number/g tissue?

L35: please move “risk” after “patient” and before “factor” in this sentence.

L127: The time frame for the early time point is unclear. “Immediately” and “within 1 day” are hard to interpret. Can you describe this is hours (<X hours)?

L159: Please add a reference for the scRNAseq study or in situ study for “given that beta-defensin 3 is epithelial-derived.”

L166-167: Natural TCRab+ and TCRgd+ cells are described in this section, but the data presented doesn’t confirm that these are the cells identified in this study. Please confirm this phenotype or remove description of “natural” TCRab+ and TCRgd+ as this is not supported. Additionally, are these cells the source of the IL-17 production and dependent on IL-1 signaling? This would help fill one mechanism gap.

L170: “indicating that cellular recruitment” This statement should be confirmed by checking that proliferation of these cells isn’t the reason for the increased abundance of cells.

L175: “their absence” I’m not sure if this is accurate. Consider revising this statement, because the data indicates that this is lower at day 1, but higher at day 2.

L183 and Figure 3a: The histology images presented here do not seem as severe as the pathology seen in Figure 1. I understand one is PAS and the other is H&E, but the tissue layers are different. Is there a spectrum of disease and disease kinetics in the IL-1RAcP-/- mouse?

L186: “However, by day 2. . .” What signals might be compensating for the IL-1 at this later time frame? IL-1 seems to be very time-period dependent throughout this manuscript. Also, in the absence of candidalysin (ece-/-) is this recruitment missing and chemokines not expressed?

L219: A clear rationale is needed for this set of experiments. If TCRab+ cell recruitment/proliferation is delayed in IL-1RAcP-/- mice and the hypothesis is that neutrophils are providing the signal to bring them in/induce proliferation, then to support this connection mechanistically you would want to design an experiment that results in restoration of this recruitment in IL-1RAcP deficient mice. Based on the presented data, it is unclear what cell types and what signals are minimally required to bring about this correction in OPC disease.

Figure 4b-d: What was seen for both mouse genotypes in the isotype control experiments for these parameters? Is this the result of Ly6G depletion and IL-1 signaling deficiency or can it be seen in IL-1 signaling deficiency alone?

Figure 4i: What would occur in a mouse treated with anakinara and Ly6G depletion? Similar to Figure 4b-d results? This experiment would help support findings above.

****

Version 1:

Reviewer comments:

Reviewer #1

(Remarks to the Author)
My concerns have been sufficiently addressed. Congratulations to the authors.

Reviewer #2

(Remarks to the Author)
The additional new data by the authors answers most of my previous queries and I think are an excellent addition to the paper. The new scRNAseq dataset is a valuable addition to the field and shed further light on the mechanisms contributing towards IL-1 mediated control of fungal infection at the oral mucosa. The reworking and new FACS data is also an important revision and I have no further queries on this.

Reviewer #3

(Remarks to the Author)
That authors have nicely addressed all my previous concerns. The new data properly addresses previous technical concerns. Moreover, the additional scRNAseq data provides new mechanistic insight to further strengthen the impact of the study.

Decision Letter:

Our ref: NMICROBIOL-25041222A

28th May 2026

Dear Julian and James,

Thank you for submitting your revised manuscript "The IL-1 Family Controls Acute Mucosal Fungal Infection and Mucosal-Systemic Dissemination." (NMICROBIOL-25041222A). It has now been seen by three of the four original referees and their comments are below. The reviewers find that the paper has improved in revision, and therefore we'll be happy in principle to publish it in Nature Microbiology, pending minor revisions to comply with our editorial and formatting guidelines.

If the current version of your manuscript is in a PDF format, please email us a copy of the file in an editable format (Microsoft Word or LaTex)-- we can not proceed with PDFs at this stage.

We are now performing detailed checks on your paper and will send you a checklist detailing our editorial and formatting requirements in about a week. Please do not upload the final materials and make any revisions until you receive this additional information from us.

Thank you again for your interest in Nature Microbiology. Please do not hesitate to contact me if you have any questions.

Sincerely,

Reviewer #1 (Remarks to the Author):

My concerns have been sufficiently addressed. Congratulations to the authors.

Reviewer #2 (Remarks to the Author):

The additional new data by the authors answers most of my previous queries and I think are an excellent addition to the paper. The new scRNAseq dataset is a valuable addition to the field and shed further light on the mechanisms contributing towards IL-1 mediated control of fungal infection at the oral mucosa. The reworking and new FACS data is also an important revision and I have no further queries on this.

Reviewer #3 (Remarks to the Author):

That authors have nicely addressed all my previous concerns. The new data properly addresses previous technical concerns. Moreover, the additional scRNAseq data provides new mechanistic insight to further strengthen the impact of the study.

Version 2:

Decision Letter:

19th June 2026

Dear Julian and James,

I am pleased to accept your Article "IL-1 family signalling drives mucosal defense against systemic Candida albicans infection" for publication in Nature Microbiology. Thank you for having chosen to submit your work to us and many congratulations.

Over the next few weeks, your paper will be copyedited to ensure that it conforms to Nature Microbiology style. We look particularly carefully at the titles of all papers to ensure that they are relatively brief and understandable.

Once your paper is typeset, you will receive an email with a link to choose the appropriate publishing options for your paper and our Author Services team will be in touch regarding any additional information that may be required. Once your paper has been scheduled for online publication, the Nature press office will be in touch to confirm the details.

You may wish to make your media relations office aware of your accepted publication, in case they consider it appropriate to organize some internal or external publicity. Once your paper has been scheduled you will receive an email confirming the publication details. This is normally 3-4 working days in advance of publication. If you need additional notice of the date and time of publication, please let the production team know when you receive the proof of your article to ensure there is sufficient time to coordinate. Further information on our embargo policies can be found here:
https://www.nature.com/authors/policies/embargo.html

After the grant of rights is completed, you will receive a link to your electronic proof via email with a request to make any corrections within 48 hours. If, when you receive your proof, you cannot meet this deadline, please inform us at rjsproduction@springernature.com immediately. You will not receive your proofs until the publishing agreement has been received through our system

Due to the importance of these deadlines, we ask you please us know now whether you will be difficult to contact over the next month. If this is the case, we ask you provide us with the contact information (email, phone and fax) of someone who will be able to check the proofs on your behalf, and who will be available to address any last-minute problems.

Acceptance of your manuscript is conditional on all authors' agreement with our publication policies (see https://www.nature.com/nmicrobiol/editorial-policies). In particular your manuscript must not be published elsewhere.

Authors may need to take specific actions to achieve compliance with funder and institutional open access mandates. If your research is supported by a funder that requires immediate open access (e.g. according to Plan S principles or the <a

href=“https://www.springernature.com/gp/open-science/us-federal-agency-compliance”> NIH public access policy) then you should select the gold OA route, and we will direct you to the compliant route where possible. Because authors warrant under our subscription licensing terms that they haven’t committed to licensing any version of their article under a licence inconsistent with the terms of our agreement – including the applicable embargo period – publication under the subscription model isn’t suitable for authors whose funders require no embargo.

If you have any questions about our publishing options, costs, Open Access requirements, or our legal forms, please contact ASJournals@springernature.com

An online order form for reprints of your paper is available at https://www.nature.com/reprints/author-reprints.html. All co-authors, authors' institutions and authors' funding agencies can order reprints using the form appropriate to their geographical region.

We welcome the submission of potential cover material (including a short caption of around 40 words) related to your manuscript; suggestions should be sent to Nature Microbiology as electronic files (the image should be 300 dpi at 210 x 297 mm in either TIFF or JPEG format). Please note that such pictures should be selected more for their aesthetic appeal than for their scientific content, and that colour images work better than black and white or grayscale images. Please do not try to design a cover with the Nature Microbiology logo etc., and please do not submit composites of images related to your work. I am sure you will understand that we cannot make any promise as to whether any of your suggestions might be selected for the cover of the journal.

You can now use a single sign-on for all your accounts, view the status of all your manuscript submissions and reviews, access usage statistics for your published articles and download a record of your refereeing activity for the Nature journals.

To assist our authors in disseminating their research to the broader community, our SharedIt initiative provides you with a unique shareable link that will allow anyone (with or without a subscription) to read the published article. Recipients of the link with a subscription will also be able to download and print the PDF.

As soon as your article is published, you will receive an automated email with your shareable link.

Congratulations once again and I look forward to seeing the article published.

With kind regards,

P.S. Click on the following link if you would like to recommend Nature Microbiology to your librarian http://www.nature.com/subscriptions/recommend.html#forms

** Visit the Springer Nature Editorial and Publishing website at www.springernature.com/editorial-and-publishing-jobs for more information about our career opportunities. If you have any questions please click here.**

Open Access This Peer Review File is licensed under a Creative Commons Attribution 4.0 International License, which permits use, sharing, adaptation, distribution and reproduction in any medium or format, as long as you give appropriate credit to the original author(s) and the source, provide a link to the Creative Commons license, and indicate if changes were made.
In cases where reviewers are anonymous, credit should be given to 'Anonymous Referee' and the source.
The images or other third party material in this Peer Review File are included in the article's Creative Commons license, unless indicated otherwise in a credit line to the material. If material is not included in the article's Creative Commons license and your intended use is not permitted by statutory regulation or exceeds the permitted use, you will need to obtain permission directly from the copyright holder.
To view a copy of this license, visit https://creativecommons.org/licenses/by/4.0/

We thank the reviewers for their constructive comments and suggestions. In response, we have performed substantial additional experiments that have strengthened the manuscript. Detailed responses to each point are provided below.

Two recurring suggestions were to further investigate the localisation of IL-1 family signalling and to strengthen the mechanistic understanding of how the IL-1 family shapes immunity during OPC. To address these points, we performed single-cell RNA sequencing of OPC-infected oral tissue. These data are presented in the revised manuscript as Figure 4, Extended Data Figure 4 and Extended Data Figure 5.

This dataset provides mechanistic insight and enabled us to define:

1. *The cellular sources of IL-1 family signals and the cell populations that respond to them.*
2. *How IL-1 family signalling promotes protective immunity during OPC.*
3. *The mechanisms by which IL-1RAcP$^{-/-}$ mice recruit neutrophils to ultimately clear infection.*

Reviewer #1 (Remarks to the Author):

This is a well-executed study that investigates the importance of IL-1R signaling in the early stages of oropharyngeal candidiasis and in controlling systemic fungal dissemination in IL-1R-deficient setting. The study underscore the role of IL-1 family on protection against fungal dissemination during OPC and on how neutrophils prevent life-threatening systemic infections in this setting.

1. A key mechanistic question is: which cell types require IL-1 signaling in the absence of neutrophils and ultimately prevent dissemination? The data show that IL-1 signaling is crucial during the early phase of infection. Testing experimentally or at least analyzing cell types where IL-1R is essential to prevent systemic candidiasis during neutropenia in OPC would substantiate this model. Such data could further help explaining why IL-1R blockade with anakinra did not result in dissemination.

We have undertaken scRNA-seq analysis of oral tissue following OPC and present this new data in Figure 4, and Extended Data Figures 4 and 5. Our new results reveal the cellular sources of IL-1 family signalling, IL-1 family communication and how the IL-1 family promote protective immunity during OPC. We show substantial signalling through IL-1α/β, IL-33 and IL-36α/β/γ, inferring the IL-1 family act in concert to coordinate protection. Therefore, we suggest anakinra blockade of IL-1R in neutropenic mice does not ablate the broader IL-1 family dependent mucosal response. We have further discussed anakinra in lines 387-392, and 405-409.

2. The finding that the ece1Δ/Δ strain, despite colonizing mucosal tissues comparably to WT, fails to disseminate systemically is intriguing. The data strongly suggest that candidalysin is essential for fungal dissemination. Is candidalysin essential once the systemic dissemination has occurred or is it necessary earlier for the penetration of the oral mucosa?

We have undertaken additional experimentation intravenously challenging control mice with AHY940 or ece1Δ/Δ and have included this data in Extended Data Figure 7c. Our results show no difference in survival between AHY940 or ece1Δ/Δ. Importantly, this new data suggests that once C. albicans breaches the mucosa and enters the systemic compartment, candidalysin is less important.

3. It will strengthen the manuscript if the authors contextualize these findings alongside previous work. For instance, PMID: 29101209 demonstrated OEC as a source of IL-1 in the OPC model triggered by candidalysin. PMID: 35296857 demonstrated a protective role of anakinra in the context of intestinal inflammation aggravated by C. albicans and candidalysin. Interestingly, in the current study, anakinra doesn't confer protection in

OPC, which highlights the complexity and context-specific nature of IL-1 signaling. Drawing these parallels could provide important perspective on how IL1R, or its blockade, can have divergent effects depending on mucosal tissue site and model used.

We have updated the manuscript to further contextualise our findings alongside both studies suggested. The added discussion can be found in the paragraphs beginning lines 339, 367, 387 and 403.

Overall, this is an insightful study that adds important mechanistic detail to the role of IL-1 signaling and candidalysin in OPC and systemic candidiasis. Addressing the points above would further strengthen its impact.

Reviewer #2 (Remarks to the Author):

This paper analyses the phenotype of IL-1RAcP-/- KO mice when challenged with the fungus Candida in a mucosal infection model. They find that the KO mice have worse control of the infection within 24 h of infection, but this resolves similarly to WT mice alongside an exaggerated immune response at 48h post infection. The authors then deplete neutrophils in their animals to mimic the main risk factor for this disease, neutropenia. Strikingly, they find the KO mice begin to succumb to the mucosal infection, with extensive dissemination to the liver and other organs which is unusual in this model. Their data therefore shows that neutrophils and IL-1 signalling co-operate for early antifungal immunity in the oral mucosa and contain the fungus.

Overall, I thought this was nice paper with new insights and was very well-presented and written. The data is high quality and reproducible. However, I felt the paper stopped short and I was hoping to learn more about the mechanisms underlying their observation.

1. For example, what cells in the mucosa are producing IL-1 and what cells are responding to it to make the neutrophil chemoattractants? How are neutrophils preventing dissemination to the liver – just by killing fungi in the mucosa? Why don't we see effects in the WT neutropenic mice in that case? Joining the dots up a bit would increase the impact of the work.

We have undertaken scRNA-seq analysis of oral tissue during OPC and present this new data in Figure 4, and Extended Data Figures 4 and 5. Our new results reveal the cellular sources of IL-1 family signalling, IL-1 family communication and how the IL-1 family promote protective immunity during OPC.

Our results show epithelia and fibroblasts are the predominant recipients of IL-1 family signalling and this stimulates these cells to express Cxcl1, Csf2, Csf3, Cxcl10, Ccl2 and Ccl20. Furthermore, we demonstrate that in the absence of IL-1 family signalling excessive mucosal damage and stress promote PGE_2 production and that this likely restores epithelial Cxcl2 and Cxcl5 expression and subsequent neutrophil infiltration.

Our findings suggest the potent infiltration of neutrophils in IL-1RAcP$^{-/-}$ mice is sufficient to clear the mucosal fungal infection, aligning with the established anti-fungal potency of neutrophils. Additionally, our data infer that neutrophils do not require IL-1 family signalling for their activation or effector function, agreeing with their reliance on PRRs once in situ.

Our results show IL-1 family signalling promotes rapid AMP production, IL-17 expression and neutrophil recruitment. While inducing neutropenia removes a key arm of the

immune response, our data suggest that AMP and IL-17 responses can restrain the fungus to the mucosal compartment despite a possible reduction in fungal clearance.

As such, I feel this paper represents an important new insight for the field and generates several outstanding remaining questions. My specific points/questions below.

2. IL-17/22/23 are all elevated in the KO mice at day 2 post-infection compared to WT. Is this linked to higher CFU in the KO mice? Is there a positive correlation between the 2 variables? Put another way, would you expect to see increased cytokines with increased CFU in WT mice, suggesting that induction of cytokines is running normally rather than an abnormal supra-induction in the absence of IL-1?

Our new scRNA-seq data suggests that while key cytokines and chemokines do recover (and in some cases potently increase) in the IL-1RAcP$^{-/-}$ model, this is due to the activation of alternative IL-1 family independent pathways and is not just controlled by infection burden.

The timely production of cytokines and chemokines is highly dependent on IL-1 family signalling, with epithelial cells and fibroblasts driving many of these signals. In contrast, we identified extensive damage and stress-associated genes at day 2 in IL-1RAcP$^{-/-}$ mice and our results suggests this promotes PGE_2 production and the recovery of key cytokines and chemokines.

3. Fig 2c; what are the FACS plots gated on? What is the population in the middle – the legend would suggest these are T-cells without a TCR? How can that be?

We have undertaken additional flow cytometry experiments to improve the T-cell gating strategy and have replaced CD3 with CD4. As a result of this updated gating, we found all CD4^{+} cells were either TCRαβ positive or TCRγδ positive. We have also updated the associated flow gating strategy, graphs and statistics to reflect the updated data from these new experiments. These can be found in Figure 2c-e, Extended Data Figure 2c-d, and Figure 5f-g.

4. The authors conclude that once T cell infiltration is established, there is no dependence on IL-1 family. However, have the authors ruled out an effect on local proliferation to determine whether there are additional influences here, rather than just on infiltration? For example, could low numbers infiltrate and then expand in the tissue, and this is just delayed in the KO mice?

We have included new data in Extended Data Figure 5c-d investigating T-cell proliferation markers and proliferation scores. This data suggests T-cells predominantly infiltrate into the tissue but a small population of these may begin to proliferate.

5. Frequency of neutrophils is shown, but it would be better to show total numbers, particularly given that there are differences in lymphocytes (and possibly proliferation?) that may skew proportions of cells as determined by flow cytometry. Quantification of their histology images may also be a help here.

There is high variability in the % of live cells between samples and between experiments, as the chemical and mechanical digestion process for tongue tissue is extremely harsh. Final cell viability is often only determined at the data analysis stage, and not while analysing the samples. Therefore, we have found that the most consistent way of presenting the data is to refer each sample to the % live cells within each sample. Despite the differences in viability, we achieved consistent populations of cells between experimental repeats using this method.

Additionally, we have included the $CD4^+$ cells as % live cells and $CD45^+$ cells as % live cells for the T-cell panels in Figure 2 and Extended Data Figure 2, and neutrophil panels in Figure 3 and Extended Data Figure 3, to alleviate concerns of possible variable upstream populations.

6. Does CARD9 play a role in the IL-1-PMN pathway? Analysis of CARD9 involvement could help explain some of the susceptibility to OPC in CARD9 deficiency and would nicely link up with previous work on IL-1-driven CARD9-dependent neutrophil recruitment in systemic infection.

This is an interesting question. However, our study focusses on the IL-1 family and their predominant function in epithelia/fibroblasts during mucosal infection. As such, investigating the role of CARD9 was outside the scope of this study.

7. Lines 327-329; slightly unfair comment given that IV infection models are good models of catheter related disease in which kidney (rather than liver) is primarily infected.

We have updated to the text to reflect the usefulness of IV Candida models in studying catheter related disease in lines 423-426.

8. Data with Anakinra in Fig 4 is somewhat at odds with the data using KO mice (and this perhaps requires more explanation/discussion). Wouldn't we expect the drug to block the IL-1 receptor and therefore most of IL-1 signalling, and would therefore mimic the

KO mice phenotype? Which aspect of IL-1 signalling isn't being blocked here and can that be leveraged to understand the mechanism in more detail?

Our new scRNA-seq dataset in Figure 4 shows substantial signalling through several members of the IL-1 family (IL-1α/β, IL-33 and IL-36α/β/γ) that predominantly involves epithelial cells and fibroblasts, but also populations in the myeloid compartment. Together, this new data suggests the IL-1 family act in concert to coordinate AMPs, IL-17 expression and neutrophil recruitment. Whilst anakinra blockade likely reduces some of this signalling and protective immunity, our results suggest compensatory activity from other IL-1 family members, and IL-1 independent pathways if the infection becomes severe. We have updated the manuscript to address this comment in the paragraph beginning line 403.

Reviewer #3 (Remarks to the Author):

In this study by Griffiths, et al the authors investigated the impact of IL-1 family cytokines to defense against mucosal Candida infection in the oral cavity. Oral Candida infections (thrush) affect millions of people worldwide. In this study the authors present exciting new data to support the conclusion that cooperation between IL-1 family cytokines and neutrophils is needed to contain Candida infection in the oral cavity and prevent dissemination. They also further identify a key role for the virulence factor candidalysin in triggering expression of IL-1 cytokines as well as being required for dissemination in immune-deficient mice (defective in both IL-1 cytokine sensing and neutrophils). The authors determined that stimulation of a human oral epithelial cell line with Candida resulted in the induction of various IL-1 family cytokines and that this induction was dependent on the virulence factor candidalysin. To investigate the impact of IL-1 family cytokines to control of oral Candida infection in vivo the authors employed IL-1RAcp-deficient mice where this deficiency results in impaired activation of multiple IL-1 family cytokines including IL-1a, IL-1b, IL-33 and IL-36 family members. In mice defective in IL-1 family responses there was a significant, but transient effect (day 2), in control of Candida infection that was resolved by day 3 of infection. At day 2 after oral infection, IL-1 deficiency affected multiple host response pathways that are known to be important for Candida containment, these included: reduced expression of antimicrobial peptides, reduced IL-17 expression and reduced recruitment of neutrophils and T cells. By day 3 after infection, control animals and IL-1RAcp-/- mice had cleared the infection (Fig 1c) which correlated with a robust infiltration of neutrophils (Fig 3). The authors tested the impact of the exuberant infiltration of neutrophils seen in IL-1RAcp-/- to control of infection by depleting neutrophils with anti-Ly6G antibodies. Strikingly, neutrophil-depleted, IL-1 deficient mice succumbed to oral infection (Fig 4a) and developed systemic infection (Figs 4b-d). They further show that candidalysin was a key factor required for dissemination where infection of IL-1-defective, neutropenic mice with a candidalysin-deficient Candida strain failed to disseminate or cause mortality. Altogether, the findings are novel, and the data presented support the key conclusions presented. My questions and recommendations for the authors are:

1. Neutrophil depletion is an important approach for the mechanistic data shown in in Figure 4. The use of anti-Ly6G antibodies is a well-accepted method to achieve neutrophil depletion in vivo. However, there are technical challenges with the approach including concerns with partial effectiveness, variable impact to neutrophil depletion in an organ-specific manner as well as technical issues with properly detecting neutrophil depletion due to antigen masking by 1A8-bound cells that appear depleted in flow cytometry especially when the staining is done with the same clone used for depletion (PMID: 31226358). Based on the data shown in Extended Data Figure 4 it appears that

the authors confirmed depletion by staining with Ly6G. Did the authors employ alternative methods to ensure proper depletion? Some options include staining with Gr1 or intracellular staining for Ly6G (PMID: 32488020).

We have undertaken further experimentation using anti-Gr1 antibody alongside Ly6G and Ly6C to more thoroughly confirm the induction of neutropenia. Our new results are presented in Extended Data Figure 6a-b. We have quantified the entire CD11b^{+} GR-1^{+} population, gated these forward and then quantified the neutrophil Ly6G^{+} Ly6C^{INT} and monocyte Ly6C^{HI} Ly6G^{-} compartments. Our new data shows that while the vast majority of GR-1^{+} CD11b^{+} cells in the isotype treated mice are neutrophils, anti-Ly6G treatment almost completely, and specifically, abolishes this neutrophil population.

2. The authors mentioned in their discussion that they "identified an influx of immature neutrophils, suggesting not only that emergency granulopoiesis can be initiated independently of the IL-1 family, but that the IL-1RAcP-/- model required substantial neutrophil infiltration to combat severe OPC infection." This is an interesting idea. Based on data shown in Extended Data Figure 3 it appears that the only metric used for identifying immature neutrophils was variable Ly6G levels. Did the authors test other important maturation markers like CXCR2 and CD101?

We have completed further experimentation to investigate neutrophil maturation state in infiltrating neutrophils during OPC. Our new data in Extended Data Figure 3g takes the entire Ly6G$^{+/HI}$ compartment and examines the expression of CD101. This new data suggests that while the neutrophil infiltration in the control mice consists of CD101^{+} mature neutrophils, both CD101^{+} and CD101^{-} cells infiltrate into the IL-1RAcP$^{-/-}$ mice.

Additionally, having investigated maturation during OPC and generated a new dataset with three repeats, we took the opportunity to improve our main neutrophil infiltration panels and have updated the plots in Figure 3C and Extended Data Figure 3c, and the associated graphs and statistical analysis in Figure 3c-e and Extended Data Figure 3b. These updated plots more clearly show neutrophil gating and have moved this population down the Ly6G axis.

3. Neutrophil depletion in IL-1RAcP-/- resulted in some intriguing effects including reduced Il-17a expression and even reduced infiltration of T cells. The authors nicely discuss how neutrophils could affect IL-17 expression based on previous work. Do they have any thoughts on how neutropenia affected T cell recruitment?

We thank the reviewer for this insightful point. As noted, our discussion already addresses potential mechanisms by which neutrophils influence IL-17A expression. While the impact of neutropenia on T cell recruitment is an important consideration, our data do not allow firm mechanistic conclusions. To avoid overinterpretation, we have

not expanded the manuscript with additional speculation, but agree this is an important area for future study.

Reviewer #4 (Remarks to the Author):

This manuscript describes the critical role for the IL-1 signaling axis in protective immune responses during mucosal candidiasis (OPC). The authors discover that IL-1 signaling (namely IL-1RAcP-depedent signaling) occurs as a result of candidalysin induced host damage during early mucosal Candida infection, which triggers IL-1 mediated recruitment of neutrophils, TCRab+, and TCRgd+ cells that help control fungal growth within the mucosa and fungal dissemination to secondary organs. The data itself is interesting, and proper controls are used in most experiments throughout the manuscript, but the study as a whole is not particularly novel (IL-1 signaling importance is candidiasis has been described) and is lacking mechanistic detail to strengthen the conclusions built on predominantly observational data. To be published in Nature Microbiology, this study requires confirmatory mechanistic analyses to concretely tie the IL-1 signaling axis to the observed findings of reduced immune cell populations to the lack of fungal control. These mechanistic analyses required for publication in Nature Microbiology may not be required for a lower impact journal.

Major Critiques:

1. Study not particularly novel and lacking mechanistic analysis required to concretely tie observations together: This manuscript although well executed and interesting is lacking a bit of novelty and impact for publication in Nature Microbiology. The importance of the IL-1 axis in candidiasis has been described by this group and others (PMID: 37478856; 29891557).

Secondly, in the present study mechanistic ties between IL-1RAcP, immune cell abundance in the oral mucosa (neutrophils, T cells, etc.), and diminished fungal control and dissemination are strictly observational connections. More concrete mechanistic study will be needed to fill the gaps between these observations and support IL-1 signaling dependence and sufficiency.

We performed scRNA-seq analysis of oral tissue following OPC and present these new data in Figure 4 and Extended Data Figures 4 and 5. These analyses identify the cellular sources of IL-1 family signalling, reveal extensive IL-1 family communication networks and signalling hubs, and define how IL-1 family signalling promotes protective immunity during OPC.

Our data show that epithelia and fibroblasts are the initial producers of IL-1 family signalling, before these signals are amplified by infiltrating myeloid cells. We demonstrate that epithelia and fibroblasts are the predominant recipients of IL-1 family signalling and activation stimulates these cells to express Cxcl1, Csf2, Csf3, Cxcl10,

Ccl2 and Ccl20, driving the recruitment of T-cells and neutrophils, and thereby mediating protective immunity.

Furthermore, we demonstrate that in the absence of IL-1 family signalling, excessive mucosal damage and stress promote PGE_2 production. This compensatory pathway drives epithelial Cxcl2 and Cxcl5 expression, resulting in robust neutrophil infiltration that ultimately resolves infection. Critically, abolishing this key arm of the immune response in IL-1RAcP$^{-/-}$ mice resulted in uncontrolled infection and dissemination of the fungi from the mucosa into the systemic compartment, primarily targeting the liver before widely disseminating.

Collectively, this study mechanistically defines the IL-1 family signalling axis during OPC. We identify candidalysin as the initiating trigger, define the primary and secondary cellular sources of IL-1 signals, map the responsive cell populations and key signalling hubs, and describe the downstream mechanisms that drive AMP, IL-17, and neutrophil-mediated protection. We also show how protective immunity can be generated in the absence of IL-1 signalling and explain why Candida dissemination occurs in neutropenic IL-1RAcP$^{-/-}$ mice. Our findings suggest that augmenting IL-1 family signalling in immunocompromised patients may represent a therapeutic strategy to prevent fungal dissemination, and that members of the IL-1 family may serve as biomarkers of severe mucosal infection.

2. More detailed analysis and discussion of IL-1RAcP is needed: Importantly, in the introduction describe why the knockout in this specific gene is needed to study IL-1 signaling defects and not other genes in mucosal candidiasis. This is described in the results (L119-121) but is missing in the introduction and is necessary to set the stage. Also, is it known whether patients with variants in IL-1RAcP gene (or other IL-1 signaling axis components) are at increased susceptibility for disease? If there are, this would greatly strengthen the clinical relevance of your research.

We have updated the introduction at lines 95-101 to better introduce our selected use of the IL-1RAcP$^{-/-}$ mice. We discuss IL-1 family gene SNPs/deficiencies in the discussion at lines 442-444. Whilst limited data suggest possible associations between IL-1 family function and patient disease, this requires extensive further investigation. Our own preliminary investigations in two fungal disease-relevant datasets suggest there may be associations but this work requires exploration in much larger cohorts alongside functional modelling of key targets. Our current study uses well established in vivo models of mucosal fungal infection that have been used to guide several outstanding clinically relevant translational discoveries.

3. Additionally, it would be important to establish the time and cell-type dependent role of IL-1RAcP during mucosal candidiasis. Which cells are known to express IL-1RAcP in

the oral mucosa and are likely to response to this IL-1 signal? How is its expression altered throughout the course of infection? Lineage tracing experiments during wildtype OPC would be invaluable to set the stage for who/what (can and is responding to these signals), when (are these signals acting), and where IL-1RAcP is acting in the context of mucosal candidiasis.

Our new scRNA-seq data in Figure 4 and Extended Data Figure 4 and 5, reveals the cellular sources of IL-1 family signals, the cellular recipients of these signals, and how the IL-1 family induce protective immunity at key signalling hubs over the course of infection.

4. Relevance to Patient Populations: When might IL-1 signaling defects be seen in the course of natural infection in humans? Is diminished IL-1 signaling present in susceptible patient populations? Is it known whether neutropenic patients have reduced IL-1 signaling or conversely are patients on anakinra, canakinumab, and rilonacept at increased risk for uncontrolled mucosal candidiasis? The introduction could benefit from the addition of the clinical relevance of exploring the IL-1 axis in disease.

We thank the reviewer for highlighting the importance of clinical relevance. Whilst we discuss how the IL-1 family have been implicated in several diseases at lines 93-94, and additionally mentioned SNPs/deficiencies possibly associated with fungal infection in the discussion at lines 442-444, there are several outstanding clinical investigations that our results in this manuscript suggest should be undertaken.

To our knowledge there are no studies investigating IL-1 family expression and/or action in immunocompromised patients with disseminated fungal infection, despite the number of IL-1 family modulating treatments currently available. As mentioned in point 2, our preliminary data from these cohorts have revealed possible associations but these require extensive further investigation before being conclusive.

The models used in our study have previously represented excellent, clinically relevant models of fungal infection. Our results suggest insufficient IL-1 family activity might represent a key clinical biomarker for severe mucosal infection, and when coupled with neutropenia, disseminated infection.

Minor Critiques:

5. Cytokine and chemokine levels throughout should be measured at the protein level.

We thank the reviewer for this important point and agree that protein expression does not always directly correlate with gene expression. However, in the context of oral

mucosal infection models, cytokine and chemokine quantification at the protein level is technically challenging and not routinely performed.

Mucosal tissue and immune cells represent a small, surface proportion of whole tongue and homogenising whole tongue results in the release of several proteinases that rapidly degrade proteins. Together, this makes attaining reproducible and accurate cytokine and chemokine protein quantification difficult.

In contrast many non-mucosal and immune cells in the tongue (muscle etc.) are very low in RNA content and thus have limited impact on gene expression data. Therefore, gene expression analyses provide a sensitive and highly reproducible readout of immune activation in this setting. This approach has been widely used in our work and by others to define functional immunological mechanisms during OPC [1-5].

6. Could you display flow data in both % total live cell and absolute number/g tissue?

When using tongue tissue there is high variability in the % of live cells between samples, and between experiments, as the chemical and mechanical digestion process is extremely harsh. Final viability is only fully determined at the data analysis stage, and not while analysing the samples. Therefore, the most consistent way of presenting the data is to refer each sample to the % live cells within each sample. Despite the differences in viability, we achieved consistent populations of cells between experimental repeats using this method.

7. L35: please move "risk" after "patient" and before "factor" in this sentence.

We have updated the manuscript with this correction, now found at line 41.

8. L127: The time frame for the early time point is unclear. "Immediately" and "within 1 day" are hard to interpret. Can you describe this is hours (<X hours)?

We have consistently used day 1 and day 2 in the Figures and so we have updated the manuscript to consistently, and more clearly, indicate day 1 or day 2 results.

9. L159: Please add a reference for the scRNA-seq study or in situ study for "given that beta-defensin 3 is epithelial-derived."

We have softened our wording and included a reference line 166-167.

10. L166-167: Natural TCRab+ and TCRgd+ cells are described in this section, but the data presented doesn't confirm that these are the cells identified in this study. Please confirm this phenotype or remove description of "natural" TCRab+ and TCRgd+ as this is

not supported. Additionally, are these cells the source of the IL-17 production and dependent on IL-1 signaling? This would help fill one mechanism gap.

We have added new data in Extended Data Figure 5a-d identifying T-cells at the exclusive source of IL-17. Our results in Figure 2b, Extended Data Figure 2b and Figure 4f show the day 1 expression of IL-17 is highly dependent on the IL-1 family, but that by day 2 IL-17 expression is restored redundantly of the IL-1 family.

11. L170: “indicating that cellular recruitment” This statement should be confirmed by checking that proliferation of these cells isn’t the reason for the increased abundance of cells.

Our new results in Extended Data Figure 5c-d alongside our results in Figure 2c-e suggest T-cells initially infiltrate before a small population may begin to proliferate. We have added a reference to this in the text at line 234-236.

12. L175: “their absence” I’m not sure if this is accurate. Consider revising this statement, because the data indicates that this is lower at day 1, but higher at day 2.

We have updated the text (now line 182) to change “absence” to “deficiency”.

13. L183 and Figure 3a: The histology images presented here do not seem as severe as the pathology seen in Figure 1. I understand one is PAS and the other is H&E, but the tissue layers are different. Is there a spectrum of disease and disease kinetics in the IL-1RAcP-/- mouse?

All histology images in Figures 1 and 3 were processed using the same protocol and equipment and include underlying muscle, endothelium, and epithelium. Histological preparation of highly muscular tissues such as the tongue is technically challenging, as the tissue tends to contract and curl after harvesting. Although careful straightening and flattening during fixation can improve section quality, consistently obtaining well-oriented sections suitable for staining remains difficult.

The day 2 IL-1RAcP$^{-/-}$ image in Figure 3 shows a highly inflammatory environment with a large amount of neutrophilic infiltration. While not the exact same tissue, the histology displayed in Figure 1 similarly shows a highly disrupted environment resulting from C. albicans infection. Our quantitative data in Figure 1C indicates all IL-1RAcP$^{-/-}$ mice have considerable CFU after 48 h. Similarly, given the subjective nature of histology images, we quantified neutrophil infiltration and identified a deficiency at day 1 in IL-1RAcP$^{-/-}$ mice before a huge recovery/infiltration of neutrophils.

14. L186: “However, by day 2. . .” What signals might be compensating for the IL-1 at this

later time frame? IL-1 seems to be very time-period dependent throughout this manuscript. Also, in the absence of candidalysin (ece-/-) is this recruitment missing and chemokines not expressed?

Our new scRNA-seq dataset presented in Figure 4, Extended Data Figure 4 and Extended Data Figure 5 reveal the initial IL-1 family-dependent signalling events that drive neutrophil recruitment. This new dataset also identifies excessive cellular damage and stress at day 2 in the IL-1RAcP$^{-/-}$ model, consistent with the high fungal burden identified at this timepoint (Figure 1c). Therefore, we investigated stress/damage associated pathways that can drive neutrophil recruitment independent of the IL-1 family and our new data suggest the production of PGE_2 (Figure 4f) alongside increased IL-17 expression (Figure 2b) drives Cxcl2 and Cxcl5, an alternative mechanism to the Cxcl1 expression seen in control mice.

In Figure 1a we show candidalysin is required for IL-1 family induction. In Figure 1e we show that in both control and IL-1RAcP$^{-/-}$ mice, candidalysin is essential for infection. Therefore, neutrophils are not required as ece1 null C. albicans cannot damage or infect.

15. L219: A clear rationale is needed for this set of experiments. If TCRab+ cell recruitment/proliferation is delayed in IL-1RAcP-/- mice and the hypothesis is that neutrophils are providing the signal to bring them in/induce proliferation, then to support this connection mechanistically you would want to design an experiment that results in restoration of this recruitment in IL-1RAcP deficient mice. Based on the presented data, it is unclear what cell types and what signals are minimally required to bring about this correction in OPC disease.

The rationale for undertaking this experiment was based on our initial surprise at the recovery of T-cells in the IL-1RAcP$^{-/-}$ model at day 2. Additionally, we were interested in the recovery of IL-17 expression in the IL-1RAcP$^{-/-}$ model at day 2 and wondered whether this was originating from T-cells or neutrophils. Recent studies have suggested neutrophils can make IL-17 and can even crosstalk with T-cells, and we mention this at lines 427-430. Although our study focusses on the IL-1 family, we have included this data as it is relevant and particularly interesting. While we show that IL-17 is derived from T-cells (Extended Data Figure 5a-b), our results suggest that possible interactions between neutrophils and T-cells may contribute to disease susceptibility/severity, particularly in neutropenic settings.

16. Figure 4b-d: What was seen for both mouse genotypes in the isotype control experiments for these parameters? Is this the result of Ly6G depletion and IL-1 signaling deficiency or can it be seen in IL-1 signaling deficiency alone?

The anti-Ly6G isotype did not reduce the presence of neutrophils (Extended Data Figure 6a-b). Isotype-treated mice therefore had normal neutrophil compartments and their responses to infection mimicked the results shown in Figure 1c. Both control and IL-1RAcP$^{-/-}$ mice restrained the infection within their mucosa and all mice had fully cleared the infection by day 3 in non-neutropenic settings (Figure 1c-d). Our data in Figure 5a show that only IL-1RAcP$^{-/-}$ mice with neutropenia succumbed to the infection, and Figure 5b-d shows that only neutropenic IL-1RAcP$^{-/-}$ mice harboured fungus in the systemic compartment. Together, these data show that IL-1 family signalling combined with neutropenia is required for dissemination. Either functional IL-1 family signalling, or neutrophils, are sufficient to restrain the infection to the mucosa.

17. Figure 4i: What would occur in a mouse treated with anakinara and Ly6G depletion? Similar to Figure 4b-d results? This experiment would help support findings above.

This experimental work is presented in Figure 5I. We show that blocking IL-1R signalling with Anakinra, or depleting IL-17A with anti-IL-17A, does not result in mucosal-systemic dissemination, but anti-IL-17A possibly worsens the mucosal infection. This is discussed in lines 387-392, 403-409, and 431-436.

References

[1] Nikou, Spyridoula-Angeliki et al. "The *Candida albicans* toxin candidalysin mediates distinct epithelial inflammatory responses through p38 and EGFR-ERK pathways." *Science signaling* vol. 15,728 (2022): eabj6915. doi:10.1126/scisignal.abj6915

[2] Conti, Heather R et al. "IL-17 Receptor Signaling in Oral Epithelial Cells Is Critical for Protection against Oropharyngeal Candidiasis." *Cell host & microbe* vol. 20,5 (2016): 606-617. doi:10.1016/j.chom.2016.10.001

[3] Taylor, Tiffany C et al. "IκBζ is an essential mediator of immunity to oropharyngeal candidiasis." *Cell host & microbe* vol. 31,10 (2023): 1700-1713.e4. doi:10.1016/j.chom.2023.08.016

[4] Kirchner, Florian R et al. "Persistence of *Candida albicans* in the Oral Mucosa Induces a Curbed Inflammatory Host Response That Is Independent of Immunosuppression." *Frontiers in immunology* vol. 10 330. 27 Feb. 2019, doi:10.3389/fimmu.2019.00330

[5] Aggor, Felix E Y et al. "Oral epithelial IL-22/STAT3 signaling licenses IL-17-mediated immunity to oral mucosal candidiasis." *Science immunology* vol. 5,48 (2020): eaba0570. doi:10.1126/sciimmunol.aba0570
